# Brain circuits for retching-like behavior

**DOI:** 10.1093/nsr/nwad256

**Published:** 2023-09-27

**Authors:** Lifang Huo, Zhimin Ye, Meiling Liu, Ziqing He, Meizhu Huang, Dapeng Li, Qian Wu, Qian Wang, Xiaoqun Wang, Peng Cao, Ji Dong, Congping Shang

**Affiliations:** School of Basic Medical Sciences, Guangzhou National Laboratory, The Fifth Affiliated Hospital, Guangzhou Medical University, Guangzhou 510799, China; Bioland Laboratory (Guangzhou Regenerative Medicine and Health Guangdong Laboratory), Guangzhou 510320, China; School of Basic Medical Sciences, Guangzhou National Laboratory, The Fifth Affiliated Hospital, Guangzhou Medical University, Guangzhou 510799, China; School of Basic Medical Sciences, Guangzhou National Laboratory, The Fifth Affiliated Hospital, Guangzhou Medical University, Guangzhou 510799, China; Bioland Laboratory (Guangzhou Regenerative Medicine and Health Guangdong Laboratory), Guangzhou 510320, China; School of Basic Medical Sciences, Guangzhou National Laboratory, The Fifth Affiliated Hospital, Guangzhou Medical University, Guangzhou 510799, China; Bioland Laboratory (Guangzhou Regenerative Medicine and Health Guangdong Laboratory), Guangzhou 510320, China; Department of Neurobiology, School of Basic Medical Sciences, Beijing Key Laboratory of Neural Regeneration and Repair, Advanced Innovation Center for Human Brain Protection, Capital Medical University, Beijing 100069, China; State Key Laboratory of Cognitive Neuroscience and Learning, IDG/McGovern Institute for Brain Research, Beijing Normal University, Beijing 100875, China; Changping Life Science Laboratory, Beijing 102299, China; State Key Laboratory of Cognitive Neuroscience and Learning, IDG/McGovern Institute for Brain Research, Beijing Normal University, Beijing 100875, China; National Institute of Biological Sciences, Beijing 102206, China; School of Basic Medical Sciences, Guangzhou National Laboratory, The Fifth Affiliated Hospital, Guangzhou Medical University, Guangzhou 510799, China; School of Basic Medical Sciences, Guangzhou National Laboratory, The Fifth Affiliated Hospital, Guangzhou Medical University, Guangzhou 510799, China; Bioland Laboratory (Guangzhou Regenerative Medicine and Health Guangdong Laboratory), Guangzhou 510320, China

**Keywords:** vomiting, nausea, nucleus of the solitary tract, Calbindin1, neural circuits

## Abstract

Nausea and vomiting are important defensive responses to cope with pathogens and toxins that invade the body. The nucleus of the solitary tract (NTS) is important for initiating these responses. However, the molecular heterogeneities and cellular diversities of the NTS occlude a better understanding of these defensive responses. Here, we constructed the single-nucleus transcriptomic atlas of NTS cells and found multiple populations of NTS neurons that may be involved in these defensive responses. Among these, we identified Calbindin1-positive (Calb1^+^) NTS neurons that are molecularly distinct from Tac1^+^ neurons. These Calb1^+^ neurons are critical for nausea and retching induced by cereulide; an emetic toxin secreted by *Bacillus Cereus*. Strikingly, we found that cereulide can directly modulate vagal sensory neurons that innervate Calb1^+^ NTS neurons, a novel mechanism distinct from that for nausea and retching induced by Staphylococcal enterotoxin A. Together, our transcriptomic atlas of NTS neurons and the functional analyses revealed the neural mechanism for cereulide-induced retching-like behavior. These results demonstrate the molecular and cellular complexities in the brain that underlie defensive responses to the diversities of pathogens and toxins.

## INTRODUCTION

In addition to escape and freezing behaviors used to avoid predators via external sensory systems (e.g. visual, auditory and olfactory cues) [1–4], vomiting and nausea are defense mechanisms against dangerous compounds or pathogens that enter the body via enteral (e.g. gastrointestinal tract) or parenteral routes, including the blood, skin, and respiratory systems [5]. These dangerous compounds can be certain toxins and chemotherapeutic drugs, which can stimulate the area postrema (AP) via blood circulation and then transmit these toxic signals to nausea and vomiting centers [6].

One of the other most common inducements of vomiting and nausea is gastrointestinal complaint triggered by bacteria-induced (e.g. *B. cereus*) food poisoning. Food poisoning-induced vomiting prompts effective and rapid expulsion of gastrointestinal pathogens, while nausea is a sensation of discomfort to avoid further feeding and future illness [6,7]. However, the neural circuits underlying food poisoning-induced vomiting and nausea remain unknown. Besides, while vomiting is always followed or preceded by nausea, little is known about the relationship between these pathways.

What's more, because of the lack of vomiting (emetic) reflex in rodents [8], most previous vomiting studies focused on cat, dog, ferret and *suncus murinus* (musk shrew) [9–12]. However, to further dig out the neural mechanism of vomiting and nausea, there is an urgent need for a mammalian animal model with clear genetic background, ease of genetic manipulation and abundant transgenic strains. The mouse is the one of the top candidates. Even though the mouse lacks a vomiting reflex, retching behavior is an alternative emetic reflex, and mice actually could exhibit some vomit-related physiological response [13]. When most gastric content is vomited out, retching usually follows. Actually, retching is the attempt to vomit without bringing anything up [14].

Staphylococcal enterotoxin A (SEA) had been revealed to have an indirect effect via immune-neuroendocrine axis (indirect pathway) for toxin-induced defensive responses [15]. There are still some scientific questions that need to be explored in depth due to molecular and cellular complexities of NTS. First, previous studies had acquired single-nucleus transcriptome of dorsal vagal complex (AP/NTS) or AP [6,16–18], while a precise NTS single-nucleus transcriptome atlas of mice was required for later unbiased screening. The molecular profiling of vagal sensory neurons had been thoroughly researched in the past few years because of its importance in sensing virous organic signals. Previous studies suggested that sensory neurons from the jugular-nodose ganglion (JNG) primarily conveyed visceral perception to the nucleus of the solitary tract (NTS) and AP [19–23]. Working as a sensory gateway, NTS detected mechanosensory and chemosensory inputs from the interoceptive nervous system via vagal sensory neurons [24]. Precise NTS transcriptome data will promote the study of neural circuits that are involved in organic signal-induced physiological responses. Second, based on molecular and cellular complexities in the brain that underlie defensive responses, did NTS exist in several neural subtypes to guarantee the safety of the internal environment? Vomiting and nausea are very important defensive behaviors, and many clinical pathogenic bacteria and toxins can induce vomiting and nausea. The neural mechanism involved may be complex and diverse. Third, vagal sensory neurons actually mediated *Bacillus cereus*-secreted cereulide via 5-HT_3_ receptors, but whether cereulide directly interacts with 5-HT_3_ receptors at vagal sensory endings or indirectly stimulates secretion of serotonin to activate 5-HT_3_ receptors is not known. Fourth, behavior observation and electrophysiological recording are not sufficient to evaluate and validate retching behavior. In addition to acquiring these physiological signals and mouth opening angle [25], intragastric pressure (IGP) [26] recording should be included. In this study, we examined the emetic effects of *B. cereus* in mice, constructed single-nucleus NTS atlas of mice and performed unbiased screening to find out the key neural population and molecular mechanism in retching-like behavior.

## RESULTS

### Chemogenetic activation of *B. cereus*-TRAPed NTS neurons induces retching-like behavior in mice

**Figure 1.**
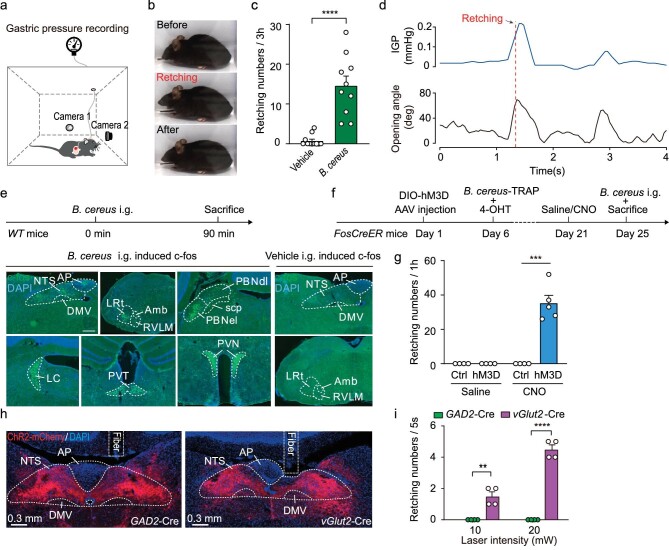
NTS plays an important role in retching-like behavior. (a) Schematic of the behavioral paradigm to monitor *B. cereus*-induced retching in mice. Gastric pressure was recorded during retching; and retching behavior was recorded by two orthogonally positioned cameras. (b) Representative images of retching induced by intragastric (i.g.) administration of *B. cereus* in mice. (c) Total number of retches for medium (vehicle) (*n* = 11 mice) and *B. cereus* (bacillus) (*n* = 10 mice) induced retching within 3 h in mice. (d) Example intragastric pressure and mouth opening angle trace in mice's retching behavior induced by *B. cereus*. (e) Experimental strategy for identifying nuclei associated with retching using *WT* mice; and brain images immunostained for a neuronal activation marker (c-Fos) 90 min after intragastric administration of *B. cereus* or vehicle. (f) Schematic showing the procedure for gaining genetic control over retching-regulating neurons. (g) Total number of chemogenetically evoked retching within 1 h in *FosCreER* mice following injections of CNO (Ctrl: *n* = 4 mice, hM3D: *n* = 5 mice) or Saline (Ctrl: *n* = 4 mice, hM3D: *n* = 4 mice). (h) Example micrographs showing ChR2-mCherry expression and the optical fiber track above ChR2-mCherry^+^ neurons in the NTS of VGlut2-ires-Cre and GAD2-ires-Cre mice. (i) *vGlut2*-ires-Cre (*n* = 4) and *GAD2*-ires-Cre (*n* = 4) mice were injected with AAVs encoding Cre-dependent ChR2-mCherry and analyzed for light-evoked retching behaviors. Data are shown as mean ± s.e.m. (error bars). The broken white lines in the section images represent boundaries of brain regions. Statistical analysis in (c) was performed using two-sided Student *t*-tests; statistical analysis in (g) and (i) was performed using two-way ANOVA (****P < 0.0001, ***P < 0.001, **P < 0.01).

*B. cereus* is a spore-forming bacterium that causes two toxin-mediated food-borne illnesses, i.e. diarrheal and emetic syndrome. Here, to test whether intragastric administration of *B. cereus* induces retching in mice, we set up two horizontal cameras to simultaneously record the time course and physiological signals of intragastric pressure (IGP) (Fig. 1a) 1 week after a latex balloon implantation (see Methods). Results showed that administration of *B. cereus*-induced retching-like behavior in mice, during which the IGP and mouth opening angle increased transiently and synchronously (Fig. 1b–d; Fig. S1b and c; and movie S1). IGP and mouth opening angle were highly correlated (Fig. S1d). Neuronal activity in the NTS, nucleus ambiguus (Amb)/rostral ventrolateral medulla (RVLM), external lateral subdivision of the parabrachial nucleus (PBNel), and stress-related brain regions were significantly elevated, as indicated by increased expression of the immediate early gene *c-Fos* (Fig. 1e). The NTS receives signals regarding gastrointestinal contents from sensory neurons in the JNG, which are conveyed by the vagus nerve. Thus, we hypothesized that the NTS may contain crucial neurons for inducing retching.

To test this hypothesis, we used the FosTRAP (*FosCreER* mice) strategy [27] to genetically activate NTS neurons that were active during *B. cereus*-induced retching-like behavior. On day 1, we injected adeno-associated virus (AAV) vector AAV-EF1a-DIO-hM3D(Gq)-mCherry into the NTS of *FosCreER* mice. On day 6, retching-like behavior in the mice was induced via *B. cereus* application and treated with 4-Hydroxytamoxifen (*B. cereus*-TRAP). After 3 weeks, the *B. cereus*-TRAPed neurons triggered robust retching-like behavior following intraperitoneal (i.p.) administration of clozapine N-oxide (CNO) compared with vehicle-TRAPed neurons (Fig. 1f and g; Fig. S1e; and movie S2). To examine the specificity and efficiency of mCherry labeling, we co-immunostained c-Fos and mCherry in the same NTS sections (Fig. S1f). Results showed that a large proportion of mCherry^+^ cells (71.3% ± 2.7%, *n* = 7 mice) were positive for c-Fos, while some c-Fos^+^ cells (46.4% ± 3.1%, *n* = 7 mice) were positive for mCherry (Fig. S1g). Chemogenetic activation of neuronal firing by CNO (10 μM) was confirmed using slice physiology (Fig. S1h). These data suggest that both the specificity and efficiency of the FosTRAP procedure for labeling *B. cereus*-associated NTS neurons are acceptable. Furthermore, to test whether the key neurons for retching were glutamatergic or GABAergic, we optogenetically activated (473 nm, 10 ms, 10 Hz, 20 mW) excitatory and inhibitory neurons in the NTS by expressing Cre-dependent ChR2 and implanting an optical fiber in *vGlut2*-ires-Cre and *GAD2*-ires-Cre mice, respectively. For *vGlut2*-ires-Cre mice, retching frequency was correlated with laser intensity, but no response was observed in the *GAD2*-ires-Cre mice, even at 20 mW (Fig. 1h and i). These results suggest that key retching neurons in the NTS are glutamatergic.

### Single-nucleus transcriptomic atlas of NTS

**Figure 2.**
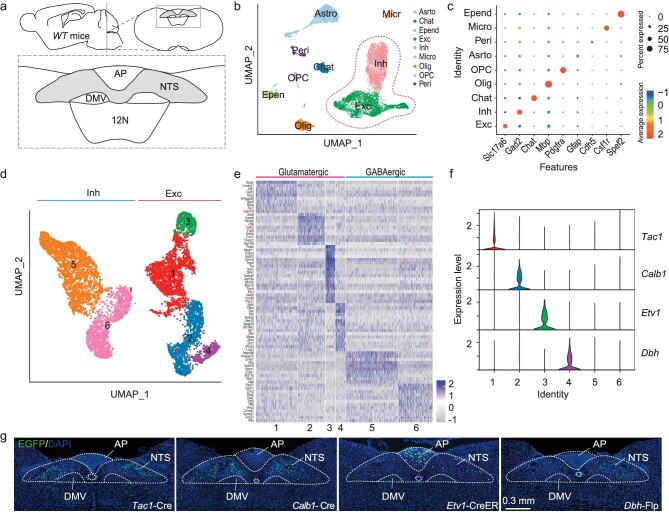
Single nucleus RNA sequencing analysis of NTS neurons. (a) Schematic illustration of the NTS location in the brainstem; NTS, nucleus of the solitary tract; AP, area postrema; DMV, dorsal motor nucleus of the vagus; 12 N, hypoglossal nucleus. (b) UMAP visualization of all NTS single cells sampled from three mice. Cell types are indicated by colors. Exc, excitatory neuron; Inh, inhibitory neuron; Olig, oligodendrocyte; OPC, oligodendrocyte precursor cell; chat, cholinergic neuron; Astro, astrocyte; Micro, microglia; Epend, ependymal cell; Peri, pericyte. (c) Dot plots showing the expression patterns of representative marker genes for each cell type, where dot size and color represent percentage of marker gene expression and the average expression level, respectively. (d) UMAP visualization showing six NTS neuronal sub-clusters including excitatory (clusters 1–4) and inhibitory neurons (clusters 5–6). Cell types are indicated by colors. (e) Heatmap of top 10 marker genes (y axis) for every sub-cluster (x axis) in NTS neurons of mice. (f) Violin plots showing the expression patterns of excitatory neuron-specific marker genes across neuronal sub-clusters. (g) Example coronal brain section showing expression of H2B-EGFP in the NTS of Cre/flp-line mice.

To identify key excitatory neurons in the NTS for retching, we first investigated cellular diversity in the NTS using single-nucleus RNA sequencing (snRNA-seq) [28] with the 10× Genomics Chromium platform [29] (Fig. 2a and Fig. S2a). After quality control, we obtained 15 604 cells from two biological replicates of wild-type (WT) mice (6 mice in total). We organized these cells into individual clusters using graph-based clustering in Seurat and batch effect correction in Harmony [30,31], and annotated them based on the classic marker genes (Fig. 2b and c, and Fig. S2b). The choline acetyltransferase positive (Chat^+^) cluster was removed, because NTS brain tissue acquired for snRNA-seq contains dorsal motor nucleus of the vagus nerve (DMV) cells and the DMV is characterized by cholinergic neurons (Fig. S2c) [32], and optogenetic activation of DMV Chat^+^ neurons failed to induce retching or any other phenotypes (Fig. S2d).

Focusing on the neuron-derived transcriptomes revealed six neuronal clusters ranging in size from 445 to 2439 (Fig. 2d). Four neuronal cell types (clusters 1–4) were glutamatergic excitatory neurons, containing 4092 neurons, while two neuronal cell types (clusters 5–6) were GABAergic inhibitory neurons, containing 4033 neurons. Signature genes in each cluster were identified, among which we selected neurotransmitter-related genes, transcription factor-related genes, and ion channels as marker genes (Fig. 2e). GO analysis results of NTS excitatory neuronal clusters implied that the genes in cluster 1 mainly located in neuroactive ligand-receptor and the genes in cluster 2 mainly located in multicellular organismal response to stress (Fig. S3). Furthermore, *Tac1, Calb1, Dbh*, and *Etv1* were selected as representative marker genes for the four excitatory neuronal clusters, respectively (Fig. 2f). We tested the expression specificity of these marker genes by injecting Cre or Flp-dependent H2B-EGFP AAV into the NTS of corresponding Cre/Flp mice (i.e. *Tac1*-ires-Cre, *Calb1*-2A-Cre, *Etv1*-CreER and *Dbh*-2A-Flp). The enhanced green fluorescent protein (EGFP)-labeled neurons were mainly distributed in the NTS (Fig. 2g). In addition, we cited and represented the mRNA *in situ* hybridization data from the Allen Brain Atlas [33] for caudal brainstem expression (Fig. S4a). The Cre/Flp mice were further used to explore the function of different neuronal subtypes in the NTS to help identify key neurons for retching.

### NTS Calb1^+^ neurons are the key neurons for retching-like behavior

**Figure 3.**
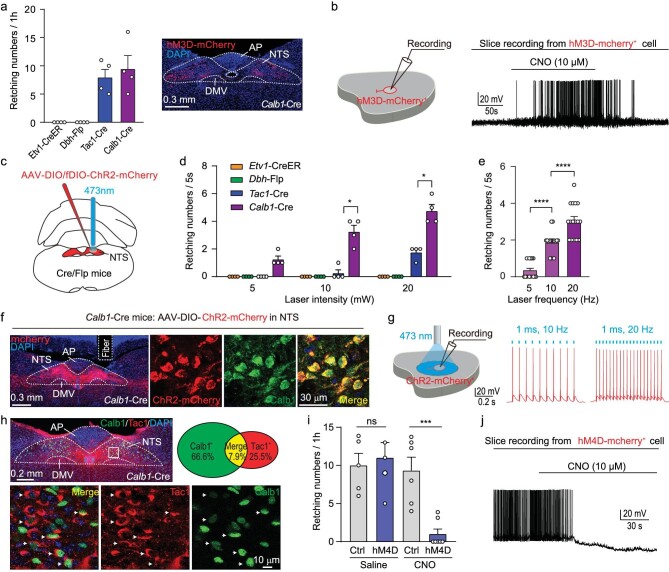
NTS Calb1^+^ neurons are the key neurons for retching-like behavior. (a) Total number of chemogenetically evoked retching within 1 h in *Calb1*-2A-Cre (*n* = 4), *Tac1*-ires-Cre (*n* = 4), *Dbh*-2A-flp (*n* = 4), and *Etv1*-creER (*n* = 4) mice following injections of CNO; and example coronal brain section showing the expression of hM3D-mCherry in the NTS. (b) Example trace of action potential firing showing the effectiveness of CNO to chemogenetically activate hM3Dq-expressing NTS neurons in acute brain slices. (c) Schematic diagram showing position of virus injection and optical fiber implantation. (d) *Calb1*-2A-Cre (*n* = 4), *Tac1*-ires-cre (*n* = 4), *Dbh*-2A-flp (*n* = 4), and *Etv1*-creER (*n* = 4) mice were injected with AAVs encoding Cre-dependent ChR2-mCherry and analyzed for light-evoked retching behaviors; for *Calb1*-2A-Cre mice, the frequency of retching during light evocation is a function of laser power. (e) Quantitative analyses of retching number of mice (*n* = 16) with optogenetic activation of Calb1^+^ NTS neurons. For *Calb1*-2A-Cre mice, the number of retching during light evocation is a function of laser frequency. (f) Example micrographs showing ChR2-mCherry expression in the NTS and the optical fiber track above ChR2-mCherry^+^ neurons in the NTS (left). Example micrographs from the NTS showing colocalization of ChR2-mCherry with the Calb1^+^ neurons (right). (g) Light-pulse trains (2 ms, 20 mW, 5 Hz, or 10 Hz) reliably evoked phase-locked spiking in ChR2-expressing NTS neurons in acute brain slices of *Calb1*-2A-Cre mice. (h) Example coronal brain section and magnified field showing colocalization of Calb1^+^ with the Tac1^+^ neurons in the NTS of *Calb1*-2A-Cre mice; the fraction of colocalization was shown on the right. (i) Total number of *B. cereus*-induced retching in mice following chemogenetic inhibition of Calb1^+^ NTS neurons (Saline: Ctrl group: *n* = 5, Test group: *n* = 4; CNO: Ctrl group: *n* = 6, Test group: *n* = 7). (j) Example trace of action potential firing showing the effectiveness of CNO to chemogenetically silence hM4Di-expressing NTS neurons in acute brain slices. Statistical analyses in (e) were performed using one-way ANOVA. Statistical analysis in (d) and (i) was performed using two-way ANOVA (****P < 0.0001, ***P < 0.001, *P < 0.05).

Next, we tested whether these four NTS excitatory neuronal clusters could induce retching-like behavior using chemogenetic and optogenetic activation in the corresponding Cre/Flp mice. AAV-Ef1α-DIO-hM3D(Gq)-mCherry was injected into the NTS of these four kinds of Cre/Flp mice. After chemogenetic activation, we found Calb1^+^ neuronal activation induced obvious retching-like behavior compared with the other three neuronal clusters (Fig. 3a; Fig. S4b and c; and movie S3). Chemogenetic activation of neuronal firing by CNO (10 μM) was confirmed using slice physiology (Fig. 3b). Then we applied optogenetic activation of the four neuronal subtypes (Fig. 3c). Consistently, optogenetic activation of Calb1^+^ neurons triggered retching-like behavior, with retching up to six times. Laser-induced retching frequency was correlated with laser intensity and laser frequency (Fig. 3d, e and f; Fig. S4c and e; and movie S4). Laser ON-OFF-ON procedure showed a reliable increase of IGP and mouth opening angle (Fig. S4f). A light-pulse train (473 nm, 2 ms, 20 mW, 10 Hz) reliably evoked phase-locked spiking activity from Calb1^+^ neurons expressing ChR2-mCherry (Fig. 3g). The immunostaining result showed that a small part of Calb1^+^ neurons are colocalization with Tac1 (7.9% ± 4.1%, *n* = 3 mice), which further implied that *Tac1* and *Calb1* belong to different subtype clusters of NTS (Fig. 3h). In AAV- Ef1α-DIO-hM3D-mCherry injected *FosCreER* mice, the co-immunostaining result showed that a majority of Calb1^+^ cells (67.9% ± 5.4%, *n* = 4 mice) were co-localized with mCherry (Fig. S5a). To quantify the proportion of colocalization of Tac1 and *B. cereus*-induced c-Fos, we injected AAV-DIO-H2B-EGFP into the NTS of Tac1-Cre mice, and administrated *B. cereus* to induce c-Fos. We found 72.1% of Calb1^+^ neurons co-express c-Fos, and 69.2% of co-Fos^+^ neurons co-express Calb1. While a very small proportion of Tac1^+^ neurons co-express c-Fos (Fig. S5b and c). Thus, these results imply that NTS Calb1^+^ neurons are sufficient to induce retching-like behavior.

To test whether NTS Calb1^+^ neurons are necessary for retching-like behavior, we chemogenetically inhibited Calb1^+^ neurons in the NTS during *B. cereus*-induced retching. Notably, *B. cereus*-induced retching was significantly suppressed by chemogenetic silencing of NTS Calb1^+^ neurons (Fig. 3i). Chemogenetic suppression of neuronal firing by CNO (10 μM) was confirmed using slice physiology (Fig. 3j). These findings suggest that *Calb1* plays an essential role in the normal function of Calb1^+^ neurons which are necessary for retching-like behavior.

### Physiological properties and projections of NTS Calb1^+^ neurons

**Figure 4.**
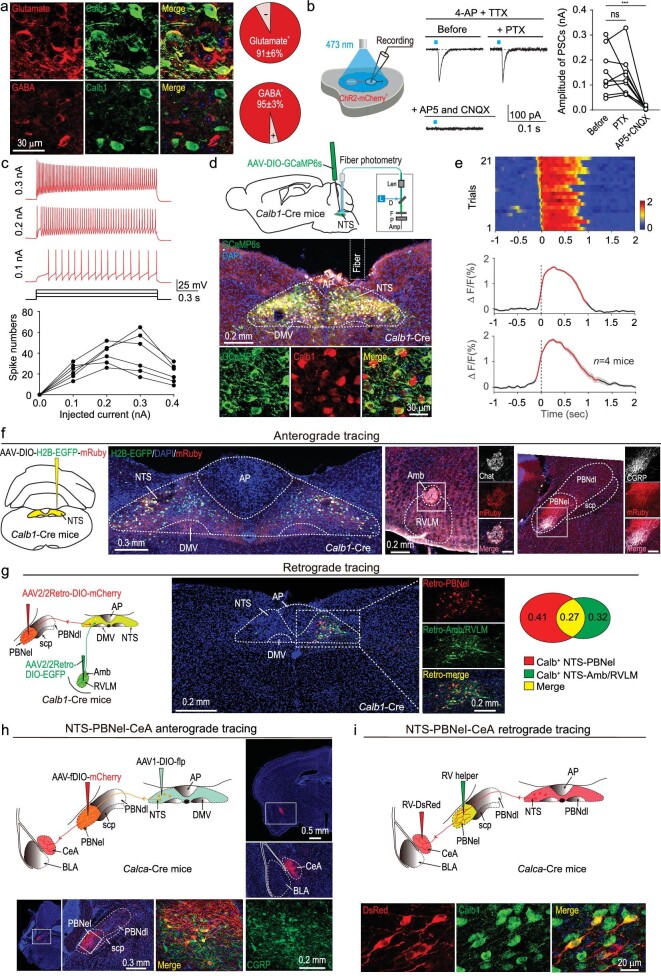
Physiological properties and projections of NTS Calb1^+^ neurons. (a) Left, micrographs showing immunostaining of Calb1 vs. glutamate (top) or Calb1 vs. GABA (bottom) in NTS. Right, quantitative analyses indicating most NTS Calb1^+^ neurons are glutamate^+^ and GABA^−^. (b) Schematic diagram showing recording of light-evoked PSCs from ChR2-mCherry-negative neurons in acute slice with NTS; and the effects of PTX and D-AP5/CNQX on light-evoked PSCs (*n* = 9 cells). (c) Spike firings of Calb1^+^ neurons in the NTS to depolarizing currents (top); and quantitative analyses of spike number as a function of current intensity (bottom) (*n* = 6 cells). (d) Schematic of the injection of AAV-DIO-GCaMP6s into the NTS of *Calb1*-2A-Cre mice, followed by optical fiber implantation above the NTS for fiber photometry recording. L, laser; D, dichroic mirror; F, filter; P, photomultiplier tube; Amp, amplifier. Example micrographs showing the optical fiber track above the GCaMP^+^ NTS neurons, which were immunohistochemically verified to be colocalization with Calb1^+^ neurons. (e) Ca^2+^ signals associated with trial of retching behavioral session. Heatmap showing 21 trials of normalized GCaMP fluorescence changes (△F/F) aligned with retching behavior induced by *B. cereus* (top); normalized GCaMP signal changes in Calb1 NTS neurons when mice's retching behavior were induced with *B. cereus*; thick lines indicate mean and shaded areas indicate s.e.m.; red segments indicate Ca^2+^ signal exceeds above 15% from baseline to peak (middle); mean Ca^2+^ transients associated with retching behavior induced by *B. cereus* for the entire test group (*n* = 4 mice) (bottom). (f) Schematic diagram and example coronal brain section showing injection of AAV-DIO-H2B-EGFP-mRuby into the NTS of *Calb1*-2A-Cre mice (left); example coronal brain section and the magnified fields (right) showing mRuby^+^ axonal projections of Calb1^+^ NTS neurons in the Amb and PBNel (right). (g) Schematic diagram of viral injection strategy to PBNel-projecting and Amb-projecting Calb1^+^ NTS neurons in the same mouse (left); example coronal brain section and the magnified fields showing the distribution of Amb-projecting (EGFP^+^) and PBNel-projecting (mCherry^+^) Calb1^+^ neurons in the NTS (middle); the fraction of EGFP-labeled (green), mCherry-labeled (red), and dually labeled (yellow) neurons in the NTS (right). (h) Schematic diagram of viral injection strategy to NTS-PBNel-CeA anterograde tracing and example coronal brain section showing the distribution of NTS-PBNel-CeA projecting. (i) Schematic diagram of viral injection strategy to NTS-PBNel-CeA retrograde tracing and example magnified fields showing the coexpression of Calb1^+^ and DsRed^+^ cells. Data are shown as mean ± s.e.m. (error bars). Statistical analysis in (b) was performed using paired *t*-tests (***P < 0.001).

To examine the physiological properties of NTS Calb1^+^ neurons, we first applied glutamate and GABA co-immunostaining with CALB1, respectively. Consistent with the above results, a large proportion of Calb1^+^ neurons (91% ± 6%, *n* = 4 mice) were glutamate^+^, while a small part (5% ± 3%, *n* = 4 mice) were GABA^+^ (Fig. 4a). We then tested the neurotransmitter types released by the Calb1^+^ neurons. We injected AAV-Ef1α-DIO-ChR2-mCherry into the NTS of *Calb1*-2A-Cre mice to express ChR2-mCherry in the Calb1^+^ neurons. In acute NTS brain slices, light pulses (473 nm, 2 ms, 20 mW) illuminating ChR2-mCherry-positive Calb1^+^ neurons evoked robust postsynaptic currents (PSCs) from ChR2-mCherry-negative NTS neurons (150 ± 30 pA, *n* = 9 neurons). The light-evoked PSCs were mediated by glutamate receptors (Fig. 4b). Finally, after labeling Calb1^+^ neurons with AAV-Ef1α-DIO-EGFP in *Calb1*-2A-Cre mice, the EGFP-labeled Calb1^+^ neurons displayed spiking activity with a slow adaptive pattern to depolarizing current injections (Fig. 4c). We next explored how NTS Calb1^+^ neurons decode the toxic signal during retching-like behavior. To monitor Calb1^+^ neuronal activity during retching-like behavior, we injected AAV-Ef1α-DIO-GCaMP6s into the NTS of *Calb1*-2A-Cre mice, then implanted an optical fiber and performed fiber photometry to record GCaMP signals. The GCaMP-labeled neurons were predominantly distributed in the NTS, and most of them were Calb1^+^ (88.9% ± 2.6%, *n* = 5 mice) (Fig. 4d and Fig. S6a). We then recorded GCaMP signals in freely moving mice during *B. cereus*-induced retching and observed a strong transient increase in GCaMP fluorescence at the onset of retching (Fig. 4e and movie S5). However, we did not observe fluorescence changes in NTS Calb1^+^ neurons expressing EGFP during retching, indicating that the recorded signals were not motion artifacts (Fig. S6b). In summary, GCaMP signals in NTS Calb1^+^ neurons are correlated with retching-like behavior.

### Calb1^NTS-PBNel^ and Calb1^NTS-Amb/RVLM^ pathways mediate retching movement and nausea, respectively

Vomiting is frequently accompanied with nausea [34], which raises the question of whether retching and nausea share the same neural circuit. To answer this question, we performed anterograde neural tracing by injecting AAV-hSyn-DIO-H2B-EGFP-mRuby into the NTS of *Calb1*-2A-Cre mice. The NTS Calb1^+^ neurons divergently projected to several brain regions, including the PBNel and Amb/RVLM complex (Fig. 4f; Fig. S7a). The PBNel contains calcitonin gene-related peptide (CGRP) neurons, which function as general alarm signals for many threats, including gastrointestinal malaise [35–37]. While the Amb/RVLM complex is located near the central pattern generator region of the brainstem [9,38]. Next, we applied retrograde tracing by injecting AAV2/2Retro-Ef1α-DIO-mCherry and AAV2/2Retro-Ef1α-DIO-EGFP into the PBNel and Amb/RVLM of *Calb*1-2A-Cre mice, respectively. The retro-labeled Calb1^+^ neurons were primarily distributed in the ipsilateral NTS and 27.43% of these neurons were double labeled (Fig. 4g), which implied that these neurons are involved in the two pathways. We also injected CTB-555 into the PBNel and Amb/RVLM of different WT mice, and the CTB-555 retro-labeled neurons were primarily Calb1^+^ (PBNel: 67.3% ± 5.2%, *n* = 4 mice; Amb/RVLM: 74.1% ± 4.5%, *n* = 4 mice) (Fig. S7b and c). We further explored the postsynaptic target of the PBNel neurons that receive NTS projection. For anterograde transsynaptic tracing, Cre-dependent AAV1-Ef1α-DIO-Flp [39,40] was injected into the NTS and Flp-dependent AAV-Ef1α-fDIO-mCherry was injected into the PBNel of *Calca*-Cre mice which express Cre in CGRP neurons [41]. The mCherry-labeled axon terminal mainly distributed in the CeA (Fig. 4h), which was confirmed by using RV-DsRed tracing. RV helper were injected in PBNel in *Calca*-Cre mice. After 2 weeks, RV-DsRed were injected into the CeA to infect the terminals of CGRP^+^ PBNel neurons. Most of DsRed^+^ neurons are Calb1^+^ (65.8% ± 7.4%, *n* = 4 mice; Fig. 4i). These findings suggest that NTS Calb1^+^ neurons mainly project to the PBNel and Amb/RVLM, and these two pathways share a small portion of Calb1^+^ neurons.

**Figure 5.**
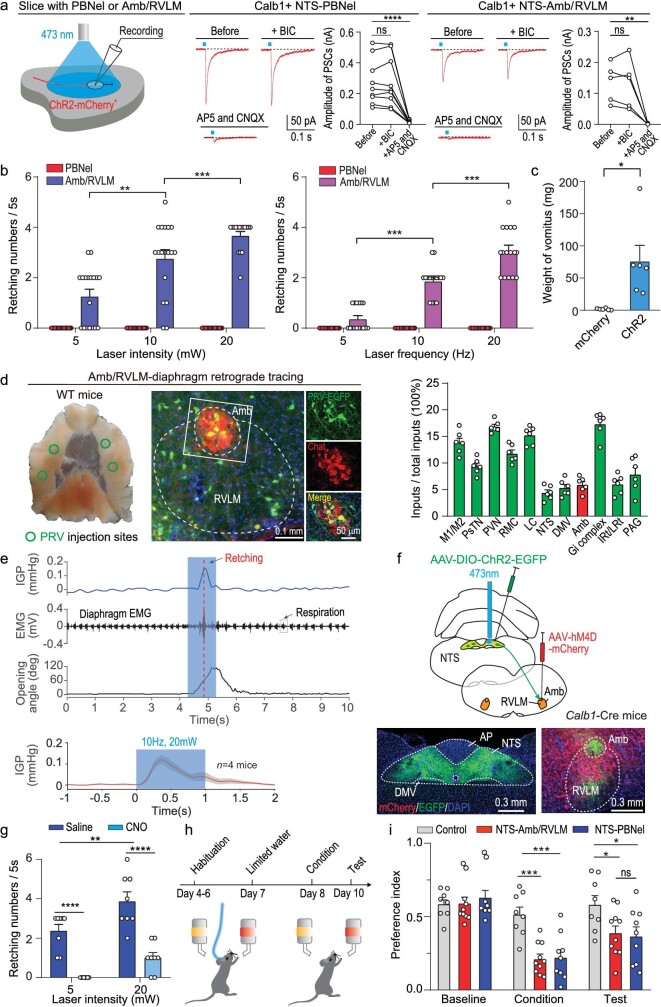
Calb1 NTS-PBNel and Calb1 NTS-Amb/RVLM pathways mediate retching movement and nausea, respectively. (a) Schematic diagram showing recording of light-evoked PSCs from ChR2-mCherry-negative neurons in acute slice with PBNel or Amb (left); and effects of PTX and D-AP5/CNQX on light-evoked PSCs recorded from ChR2-mCherry negative neurons in the PBNel (*n* = 9 cells) and Amb (*n* = 6 cells) (right). (b) Quantitative analysis of retching number of mice with optogenetic activation of the NTS-Amb (*n* = 18) or NTS-PBNel (*n* = 18) pathway. For NTS-Amb pathway, the frequency of retching during light evocation is a function of laser power and intensity. (c) The vomitus weight was analyzed (mCherry group: *n* = 6 mice, ChR2 group: *n* = 6 mice). (d) Schematic diagram of PRV viral injection site to diaphragm; and the example coronal brain section and the magnified fields showing the distribution of retrogradely labeled Amb neurons (left); fraction of total PRV-labeled cells in different brain regions projecting to the diaphragm. Note that data in (d) were normalized by dividing the total number of PRV^+^ cells in these brain regions (right). (e) Schematic of intragastric pressure (IGP), EMG and mouth opening angle trace when photostimulation of the Calb1NTS-Amb/RVLM pathway induces retching behavior in mice (*n* = 4 mice). (f) Schematic diagram showing position of virus injection and optical fiber implantation and an example coronal brain section showing the expression of ChR2-EGFP or hM4D-mCherry. (g) Total number of light-evoked retching in mice following injection of CNO to inhibit Amb neurons (Saline: *n* = 8 mice, CNO: *n* = 8 mice). (h) Schematic showing the procedure of behavioral assay for CFA. (i) Quantitative analyses of preference (Control: *n* = 8 mice, NTS-Amb: *n* = 10 mice, NTS-PBN: *n* = 6 mice). Data are shown as mean ± s.e.m. (error bars). Statistical analysis in (a) was performed using paired *t*-tests; statistical analysis in (b) was performed using two-way ANOVA; statistical analysis in (c) was performed using two-sided Student *t*-tests; statistical analyses in (g) and (i) were performed using two-way ANOVA (****P < 0.0001, ***P < 0.001, **P < 0.01, *P < 0.05).

To explore the synaptic targets of the Calb1^NTS-PBNel^ and Calb1^NTS-Amb/RVLM^ pathways, we performed PSC recordings in acute brain slices. AAV-Ef1α-DIO-ChR2-mCherry was injected into the NTS of *Calb1*-2A-Cre mice. In acute brain slices, light pulses (473 nm, 2 ms, 20 mW) illuminating ChR2-mCherry^+^ axon terminals from the NTS Calb1^+^ neurons evoked robust postsynaptic currents (PSCs) in PBNel neurons (274 ± 50 pA, *n* = 9 neurons), and the same light stimulation evoked slightly weak PSCs in Amb/RVLM neurons (134 ± 28 pA, *n* = 9 neurons; Fig. 5a). The light-evoked PSCs in PBNel and Amb/RVLM neurons were both mediated by glutamate receptors.

We then examined the function of these two neural pathways using optogenetic activation. AAV-Ef1α-DIO-ChR2-mCherry was injected into the NTS of *Calb1*-2A-Cre mice, and optical fibers were implanted above the Amb/RVLM and PBNel, respectively. Optogenetic activation of the Calb1^NTS-Amb/RVLM^ pathway elicited retching-like behavior, whereas activation of the Calb1^NTS-PBNel^ pathway did not induce any overt behavioral phenotypes. Repeated activation of the Calb1^NTS-Amb/RVLM^ pathway caused forceful vomiting in satiated mice with intragastric administration of dragon fruit juice (Fig. 5b and c; Fig. S8a; and movie S6; see Methods). Retching is controlled by respiratory muscles, which consist of intercostal muscle, diaphragm muscle, and abdominal muscle [42]. To determine which muscles are targeted by the Calb1^NTS-Amb/RVLM^ pathway, we performed pseudorabies virus (PRV) vector PRV-GFP retrograde tracing by injecting PRV-GFP (PRV 531) into these muscles [43]. Only retro-labeled GFP^+^ neurons from the diaphragm muscle were distributed in the Amb/RVLM complex (Fig. 5d and Fig. S7d). We applied diaphragm muscle electromyography (EMG) and IGP recordings during optogenetic activation of the Calb1^NTS-Amb/RVLM^ pathway and observed transient increases in both EMG and IGP correlated with retching (Fig. 5e; Fig. S8b–e; and movie S7), suggesting that the Calb1^NTS-Amb/RVLM^ pathway was responsible for diaphragm muscle contraction to induce IGP.

As activation of the Calb1^NTS-Amb/RVLM^ pathway induces retching-like behavior, we wondered whether this pathway is necessary for retching. We chemogenetically silenced the Amb/RVLM complex, and optogenetic activation of the cell body of NTS Calb1^+^ neurons (Fig. 5f). Compared to saline i.p. injection, CNO i.p. injection nearly abolished retching-like behavior (Fig. 5g and Fig. S8f). Thus, these results imply that the Calb1^NTS-Amb/RVLM^ pathway is sufficient and necessary for *B. cereus*-induced retching.

Previous studies have confirmed that PBNel CGRP neurons mediate conditioned flavor aversion (CFA) as a general alarm signal [35,44], and the CFA test is a typical paradigm to reflect ‘nausea’ [6,45]. As such, we hypothesized that the Calb1^NTS-PBNel^ may signal gastrointestinal malaise from *B. cereus* and establish CFA. To test this hypothesis, we optogenetically activated the Calb1^NTS-PBNel^ and Calb1^NTS-Amb/RVLM^ pathways and performed CFA to determine whether these two pathways promote CFA (Fig. 5g). In the memory test stage, both the NTS-PBNel group and the NTS-Amb/RVLM group exhibited conditioned aversion (Fig. 5h and i). There are two possible reasons for this result. (1) Activation of Calb1^NTS-Amb/RVLM^ pathway-induced retching movement may disturb drinking behavior. (2) Based on retrograde tracing of the two pathways, retro-labeled Calb1^+^ neurons showed 27.43% colocalization (Fig. 4g), which may lead to partial co-activation of both pathways (i.e. activation of one pathway may activate some neurons in the other pathway). Together, these results suggest that the Calb1^NTS-Amb/RVLM^ pathway is responsible for retching movement to expel toxic foods, while the Calb1^NTS-PBNel^ pathway plays a role in inducing nausea and establishing CFA.

### JNG sensory neuronal subtype directly transmits cereulide toxin signal to NTS Calb1^+^ neurons to induce retching-like behavior

Finally, we wondered how signals of the *B. cereus* emetic toxin (cereulide) are transmitted to the NTS from the digestive tract [12,46]. Based on the result that intragastric administration of cereulide could induce retching-like behavior (Fig. S9a and b), we performed whole-cell recordings from cultured JNG sensory neurons and identified three types (type I-responseless, type II-inhibited and type III-activated) according to their reactivity to cereulide (Fig. 6a). After recording, we performed SMART-seq2 [47] single-cell RNA sequencing (scRNA-seq) to obtain the expression patterns of these neurons (Fig. S10). We applied rabies virus (RV) vector RV tracing to identify the retro-labeled JNG sensory neurons and performed scRNA-seq to obtain their expression profiles [48,49]. RV helper AAVs were injected into the NTS of *Calb1*-2A-Cre mice, followed by RV-DsRed injection two weeks later (Fig. 6b and c). The retro-labeled neurons were mainly located in the PVN, CeA, and JNG (Fig. 6d and Fig. S11).

**Figure:**
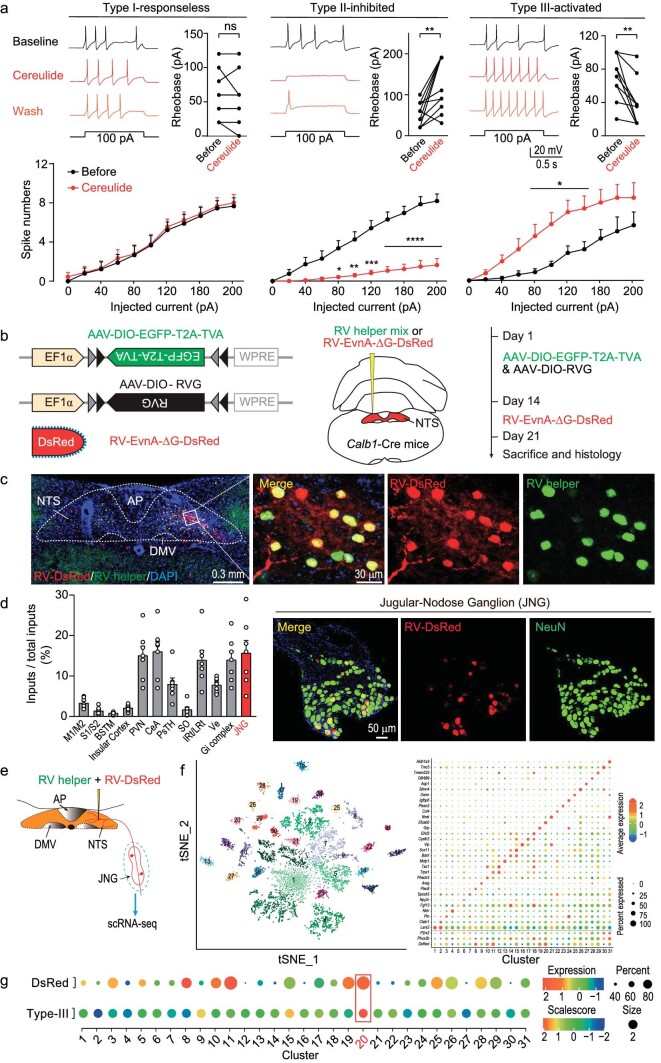
JNG sensory neuronal subtype transmits cereulide toxin signal to NTS Calb1^+^ neurons to induce retching-like behavior. (a) Example of action potentials response to 100 pA current injections in distinct types of JNG neurons that were untreated or treated with 0.4 ng/μL cereulide and washing. Effects of cereulide on the rheobase (type III-activated: *n* = 10 cells, Type II-inhibited: *n* = 12 cells, Type I-no response: *n* = 8 cells) and the excitability with increasing current injection of three distinct types of JNG neurons (Type I-no response: *n* = 9 cells, Type II-inhibited: *n* = 12 cells, type III-activated: *n* = 9 cells). (b) Schematic diagram showing the strategy for monosynaptic retrograde tracing of Calb1^+^ NTS neurons by using a combination of AAV and RV. (c) Example coronal brain section and magnified field showing the dually labeled starter cells in the NTS of *Calb1*-2A-Cre mice. (d) Fractions of total RV-labeled cells in different brain regions monosynaptically projecting to the Calb1^+^ NTS neurons. Note that data in (d) were normalized by dividing by the total number of DsRed^+^ cells in these brain regions (left); example micrographs showing colocalization of DsRed^+^ cells with the neuron in JNG (right). (e) Schematic diagram showing RV-labeled cells in JNG monosynaptically projecting to the Calb1^+^ NTS neurons. (f) t-SNE visualization of the JNG clusters (left) and dot plot showing the expression patterns of representative marker genes for each cell type, where dot size and color represent percentage of marker gene expression and the average expression level, respectively (right). (g) Dot plot of DsRed expression of DsRed (up) and the signature scores of type III neurons (bottom) across all the 31 JNG neuronal clusters. Data are shown as mean ± s.e.m. (error bars). Statistical analysis in (a) was performed using paired *t*-tests; and two-way ANOVA (****P < 0.0001, ***P < 0.001, **P < 0.01, *P < 0.05), NS, not significant (*P* > 0.1).

Then we acquired RV-tracing JNG and performed 10X Genomics scRNA-seq (Fig. 6e and Fig. S12a). After QC, 15 698 cells were retained and organized into individual clusters, consistent with their unique qualities (Fig. S12b–d). We focused on the JNG sensory neurons from Fig. S12b, and the JNG sensory neuron-derived transcriptomes revealed 31 neuronal clusters ranging in size from 57 to 732, among which DsRed labeled several clusters, and signature genes in each cluster were identified (Fig. 6f). Next, we used the marker genes of type III-activated neurons to calculate their signature score across all 31 clusters, and found that type III neurons were similar to the DsRed^+^ cluster 20 of JNG sensory neurons (Fig. 6g and Fig. S13a). Furthermore, we performed difference expression analysis and signature genes in each cluster were further identified for JNG type III neurons. Htr3a, a subtype of 5-HT_3_ receptor, is highly expressed in cluster 20 (highlight in red, Fig. S13b)). A previous study suggested that cereulide-induced vomiting is mediated by 5-HT_3_ receptors [12]. However, whether cereulide directly interacts with 5-HT_3_ receptors at vagal sensory endings or indirectly stimulates secretion of serotonin to activate 5-HT_3_ receptors is not known. These results suggested that cereulide may directly innervate 5-HT_3_ receptor positive neurons.

## DISCUSSION

The nervous system owns robust and diverse risk sensing neurons to avoid pathogen or toxin intrusion into the internal environment. Distinct neuron subtypes detect different toxins, but share the same effective responses (e.g. nausea, retching, and vomiting). Our previous study found Tac1^+^ NTS neurons mediate SEA-induced retching-like behavior. Based on our developed *B. cereus*-induced retching-like paradigm and constructed single-nucleus transcriptomic atlas of NTS in mice, we found a new neuron subtype involved in retching-like behavior induced by *B. cereus*-mediated food poisoning and uncovered therein a neural mechanism. We summarized the main findings and showed the neural mechanism of this study with a cartoon (Fig. S14).

### Single-nucleus transcriptomic atlas of NTS

The brain receives multiple critical sensory signals (chemicals and mechanical forces) from internal organs via the vagal nerve. Vagal sensory axons primarily target the NTS, a sensory gateway in the brainstem. The vagal sensory map had well-defined neurons, including JNG and NTS While NTS lacked a molecular defined neuronal subtype we obtained original NTS snRNA-seq data. Considering the critical role of the NTS in sensing the physiological status of internal organs, our sequencing database should facilitate further studies on how the central nervous system detects other microorganisms and pathogens transmitted by the JNG-NTS pathway, such as respiratory infection [50,51], especially for the SARS-CoV-2 virus.

### NTS Calb1^+^ and Tac1^+^ neurons are distinct subtype neurons for retching-like behavior

Based on our constructed single-nucleus NTS transcriptomic atlas of NTS, we performed optogenetic and chemogenetic screening to find which cluster is the key neuron for retching-like behavior. Actually, the signature genes *Tac1* and *Calb1*, which are the different expression genes of cluster 1 and cluster 2, respectively, both can trigger retching-like behavior. Besides, the immunostaining result showed that a small part of Calb1^+^ neurons are colocalized with *Tac1* (7.9% ± 4.1%), which further implied that *Tac1* and *Calb1* belong to a different subtype cluster of NTS. Because *Tac1* and *Calb1* belong to different clusters, this implied that Tac1^+^ and Calb1^+^ neurons may be involved in the detection of distinct toxins [15]. Catecholamine neurotransmission was considered to play a crucial involvement in nausea and vomiting by evaluating kaolin intake [52], and we observed activation of tyrosine hydroxylase (TH)-positive neurons induced retching behavior in TH-ires-CreER mice (data not shown), while we had not observed vomiting behavior after activating Dbh^+^ NTS neurons (Fig. 3a and d). These neurons may be involved in nausea response, but we did not perform CFA testing for other Flp/Cre line mice except for *Calb1*-2A-Cre mice.

### NTS Calb1^+^ neurons as the key neuronal subtype for cereulide-induced retching-like behavior

Based on the NTS snRNA-seq data and unbiased screening of NTS neuronal clusters, we identified Calb1^+^ neurons as the key neuronal subtype for retching-like behavior. Except for JNG sensory neurons, monosynaptic inputs tracing data implied that Calb1^+^ neurons also received direct projections from the motor, sensory and insular cortices, vestibular system, AP and fastigial nucleus (Fig. 6d and Fig. S11) [53,54], which are important brain regions implicated in vomiting and nausea [5,34,53,55]. NTS Calb1^+^ neurons are probably the regulatory center of retching. It remains to be seen how NTS Calb1^+^ neurons integrate the nervous system signals to induce retching.

Working as calcium (Ca^2+^) buffering, CALB1 play a critical role in the Ca^2+^-dependence of many intracellular processes, especially for Ca^2+^-dependent neurotransmitter release [56,57]. The suppressed expression level of CALB1 mediated hippocampal function and cognition [58,59]. AP could detect blood-borne toxins and convey neuronal signals to CGRP neurons in PBNel to induce nausea and did not cause retching-like behavior [6,54], while Calb1^NTS-Amb/RVLM^ pathway activation induced forceful vomiting behavior in satieted mice (movie S6). CALB1 mainly express in NTS but not AP (Fig. 2g), which implies that the NTS-PBNel pathway is parallel to the AP-PBNel pathway. Whether suppressed CALB1 levels lead to Ca^2+^-dependent neurotransmission, the dynamic characteristic changes of neurotransmitter release remains to be analyzed.

### Brain circuits to coordinate *B. cereus*-induced retching-like behavior

JNG sensory neurons transmitted *B. cereus*-secreted cereulide toxin signals from the digestive tract to NTS Calb1^+^ neurons, which then separated these signals into divergent pathways. One pathway projected to the Amb/RVLM and the other pathway projected to the PBNel and forward to the CeA. The Amb/RVLM complex is located near the central pattern generator region of the brainstem, which is responsible for breathing generation via abdominal muscle, intercostal muscle and diaphragm muscle [9,38]. Our PRV retrograde tracing data implied Calb1^NTS-Amb/RVLM^ pathway innervates diaphragm muscle contraction to initiate vomiting of toxic food (Fig. 5d). Calb1^NTS-PBNel^ pathway activation induced the nausea response via CFA analysis. CGRP neurons in PBNel may play a critical role in nausea.

### Direct activation of JNG neurons by cereulide

A previous study suggested that cereulide-induced vomiting is mediated by 5-HT_3_ receptors. However, whether cereulide directly interacts with 5-HT_3_ receptors at vagal sensory endings or indirectly stimulates secretion of serotonin to activate 5-HT_3_ receptors is not known. This recent study found out the indirect pathway of activation 5-HT_3_ receptors for staphylococcal enterotoxin A (SEA) [15], while the study suggested that cereulide can directly activate Htr3a positive JNG neuronal clusters. The cumulative effect of cereulide may be necessary for retching-like behavior which accounts for the delayed arrival of defensive response. However, whether the direct neural mechanism of cereulide-induced retching was mediated by 5-HT_3_ receptor remained to be further dissected.

## MATERIALS AND METHODS

### Animals

The *Calb1*-2A-Cre, *Tac1*-ires-Cre, *Dbh*-2A-Flp, *Etv1*-CreER, *vGlut2*-ires-Cre, *GAD2*-ires-Cre, *Chat*-Cre and *FosCreER* (Fos-2A-iCreER) mouse lines were imported from Jackson Laboratory (JAX Mice and Services). Mice were housed at room temperature (23 ± 1°C) with a stable humidity (50% ± 5%) and free access to food/water on a 12 h/12 h light/dark cycle. Because we did not observe a statistical difference between male and female mice for *B. cereus*-induced retching-like behavior (Fig. S1a), almost the same amount of male and female mice were used in each experiment including control and test groups. All experimental procedures were conducted following protocols approved by the Administrative Panel on Laboratory Animal Care at the Guangzhou laboratory (Guangzhou, China).

### *B. cereus*-induced retching behavior

The stored strains were resuspended with 300 μL medium and returned to room temperature. Mice with acute gastritis were intragastrically administrated *B. cereus* or a vehicle with 10 μL/g once an hour for a total of 3 hours. The retching behaviors were recorded by two orthogonally positioned cameras (50 frames/s; Point Grey Research, Canada).

### Measuring vomitus of retching mice

AAV-DIO-ChR2-mCherry or AAV-DIO-mCherry were stereotaxically injected into the NTS of Calb1-2A-Cre mice as test or control groups, respectively. After AAV injection and fiber implantation, the mice were housed individually for 3 weeks. On the day of experiment, the test mouse was allowed to explore an area (20 cm × 20 cm, square open field) for 15 minutes. Mice were slowly given dragon fruit juice (1 mL/10 g, approximately 2.5 mL/each mouse) by gavage and then returned to the cage and had a rest for 10 min. Before laser stimulation, the residual dragon fruit juice around the mouths of the mice was cleaned up, and then a laser stimulation protocol (10 Hz, 10 mW, 10 ms, 20 s) was given to induce intense retching behaviors in the mice. The strong light evoked the mice to vomit some of the dragon fruit juice. Red dragon fruit juice was more obvious than other foods. The weight of the vomitus can be calculated by taking a clean paper and weighing it, then wiping the vomitus with the paper and weighing it again.

### Intragastric administration for mice

With one hand, the mouse's tail was lifted onto a rough surface, while the other hand grasped the mouse and fixed the head, trunk and tail of the mouse so that the mouse's head and trunk were kept in a straight line and in a head-high-tail-low position. The end of the 11-gauge gavage tube was inserted through the corner of the mouse's mouth, and slowly inserted into the stomach along the oesophagus, feeling no resistance during the insertion of the gavage tube. The drug was then slowly injected, and if the mouse did not respond, the remaining drug was rapidly injected. Finally, the tube was slowly removed and the mice were returned to the cage.

## Supplementary Material

nwad256_Supplemental_Files

## Data Availability

Sequencing data is deposited in the Genome Sequence Archive (GSA) database (GSA accession number: CRA007790). Code is available on request. All other data are available in the main text or the Supplementary materials.
