# Supplementary material for: Brain circuits for retching-like behavior
Source: Natl Sci Rev. 2023 Sep 27;11(1):nwad256. doi: 10.1093/nsr/nwad256 (PMC10824557; doi:10.1093/nsr/nwad256)
Supplement: nwad256_Supplemental_Files [file nwad256_supplemental_files.zip › Supplementary Figure S1-14 legends.docx]

**Supplementary information**

**Supplementary figure legends**

**Supplementary Figure S1. *B.cereus* induced retching-like behavior in mice.**

**a** Statistical analysis of *B.cereus*-induced retching behavior in male (n=4 mice) and female mice (n=4 mice).

**(b-c)** Average curve and statistical plot of peak of IGP (b) and opening angle (c) using *B.cereus* to induce retching-like behavior in mice (n=6 mice, Trials=18).

**d** Correlation analysis of IGP and opening angle (n=18 Trials).

**e** Total numbers of medium (n=6 mice) or B. cereus (n=6 mice) evoked retching within 3 h in *FosCreER* mice.

**f** Representative images showing the expression of hM3D-mCherry in NTS and the colocalization c-Fos (green)/hM3D-mCherry (red).

**g** Quantitative analysis of the proportion of co-localized neurons of c-Fos^+^ and mCherry^+^ in mCherry^+^ and c-Fos^+^ neurons respectively.

**h** Schematic diagram showing whole-cell recording of mCherry^+^ NTS neurons in acute slices; And the example traces of action potential firing showing the effectiveness of CNO to chemogenetically activity hM3Dq-expressing NTS neurons in acute brain slices. CNO was dissolved in artificial cerebrospinal

fluid (ACSF) (10 μM) and perfused to the brain slices.

Data are shown as mean± s.e.m. (error bars). The broken white lines in the section images represent boundaries of brain regions. Statistical analyses in a, b, c, e, g were performed using two-sided Student t-tests (***P<0.001).

**Supplementary Figure S2. Single-nucleus NTS UMAP analysis of mice.**

**a** Schematic illustration for preparation of single-nucleus RNA.

**b** UMAP visualization of the expression patterns of cell type-specific marker gene; Grey to red indicates low to high expression levels, respectively.

**c** UMAP visualization of the expression patterns of NTS neuronal marker genes.

**d** Example micrographs showing ChR2-mCherry expression and the optical fiber track above ChR2-mCherry^+^ neurons in the NTS of Chat-cre mice and analyzed for light-evoked retching behaviors of VGlut2-ires-Cre (n=4 mice), GAD2-ires-cre (n=4 mice), Chat-cre mice (n=4 mice).

Data are shown as mean ± s.e.m. (error bars). Statistical analysis in d was performed using two-way ANOVA (****P < 0.0001).

**Supplementary Figure S3. GO analysis results of NTS excitatory neuron clusters based on snRNA-Seq data.**

**Supplementary Figure S4. Validation of retching behaviour induced by activation of Etv1^+^, Dbh^+^, Tac1^+^ and Calb1^+^ neurons in the NTS, respectively.**

**a** RNA in situ hybridization data from the Allen Brain Atlas showing expression of signature genes in NTS.

**b** Example coronal brain section showing the expression of hM3D-mCherry in the NTS of *Etv1*-creER mice, *Dbh*-2A-flp, *Tac1*-ires-cre.

**c** Quantitation the retching numbers in mice *Etv1*-creER (n=4), *Dbh*-2A-flp (n=4), *Tac1*-ires-cre (Saline: Ctrl n=4, hM3D n=4; CNO: Ctrl n=4, hM3D n=6) and *Calb1*-2A-cre (Saline: Ctrl n=4, hM3D n=4; CNO: Ctrl n=4, hM3D n=5) with and without chemogenetically activation of ETV1^+^, Dbh^+^, Tac1^+^ and Calb1^+^ NTS neurons.

**d** Example micrographs showing ChR2-mCherry expression and the optical fiber track above ChR2-mCherry+ neurons in the NTS of *Etv1*-creER, *Dbh*-2A-flp, *Tac1*-ires-cre mice.

**e** *Etv1*-creER (n=4), *Dbh*-2A-flp (n=4), *Tac1*-ires-cre (n=4) and *Calb1*-2A-cre (n=4) mice were injected with AAV-DIO-mcherry in NTS as control and analyzed for light-evoked retching behaviors; For *Calb1*-2A-cre mice who were injected with AAV-DIO-chR2-mcherry in NTS , the frequency of retching during light-evoked is a function of laser power.

**f** Schematic of Intragastric pressure (IGP) and mouth opening angle trace during the activation of NTS Calb^+^ neurons to induce retching behavior in mice using a continuous photostimulation protocol (Off-On-Off-On).

Data are shown as mean± s.e.m. (error bars). The broken white lines in the section images represent boundaries of brain regions. Statistical analyses in c, e were performed using two-way ANOVA.

**Supplementary Figure S5. The neurons activated by the retching behavior induced by *B. cereus* are mostly Calb1^+^ NTS neurons.**

**a** Example micrograph and magnified fields from the NTS of *FosCreER* mice injected with AV-DIO-hM3D-mCherry in NTS showing colocalization of hM3D-mCherry with the Calb1^+^ neurons scale bar=0.2mm (Left) ; Quantitative analysis of the proportion of co-localized neurons in Calb1^+^ neurons (n=7 Fields).

**b** Example micrograph and magnified fields showing the colocalization of c-Fos induced by *B.cereus* with Calb1 and H2B-EGFP in *Tac1*-cre mice injected with AAV-DIO-H2B-EGFP in NTS scale bar=0.2mm.

**c** Statistical proportional analysis of Tac1^+^, Calb1^+^, Calb1^+^/ Tac1^+^ and Calb1^-^/Tac1^-^ neuron.

**d** Quantitative analysis of the proportion of co-localized neurons of c-Fos^+^ and Calb1^+^ in Calb1^+^ and c-Fos^+^ neurons respectively (n=5 fields) (Left); And the proportion of co-localized neurons of c-Fos^+^ and Tac1^+^ in Tac1^+^ and c-Fos^+^ neurons respectively (n=5 fields) (Right).

**Supplementary Figure S6. Fiber photometry recording of NTS Calb1^+^ neurons.**

**a** Quantitative analysis of the proportion of co-localized neurons in Calb1^+^ and

GCaMP^+^ neurons respectively (n=7 fields).

**b** EGFP signal when mice were induced retching behavior with *B. cereus*.

**Supplementary Figure S7. Divergent projections of Calb1^+^ NTS neurons.**

**a** Example coronal brain sections of *Calb1*-2A-Cre mice with EGFP-Syb2 expression in the NTS and EGFP-Syb2^+^ axon terminals expression in different brain regions. The boundary of the Amb was delineated according to the immunofluorescence of vacht (red).

**b** Schematic diagram showing unilateral injection of CTB-555 into the Amb of the WT mice; And example micrographs showing CTB-555^+^ cells in the NTS were predominantly positive for Calb1.

**c** Schematic diagram showing unilateral injection of CTB-555 into the PBNel of the WT mice; And example micrographs showing CTB-555+ cells in the NTS were predominantly positive for Calb1.

**d** Eight micrographs showing the projection fields of the diaphragm neurons where PRV viral was multi-point injected. M1: primary motor cortex; M2: secondary motor cortex; PSTN: Parasubthalamic nucleus; PVN: Paraventricular hypothalamic nucleus; RMC: Red nucleus, magnocellular part; LC: locus coeruleus; NTS: Nucleus of the solitary tract; DMV: Dorsal motor nucleus of vagus; Amb: Ambiguus nucleus; Gi complex: Gigantocellular reticular nucleus; IRt: intermediate reticular nucleus; PAG: Periaqueductal

gray.

**Supplementary Figure S8.** **Calb1^NTS-Amb/RVLM^ pathways mediate retching movement.**

**a** Quantitative analysis of retching number of mice with optogenetic activation of the NTS-Amb or NTS-PBNel pathway (Ctrl-PBNel n=6mice, Ctrl-Amb/RVLM n=6 mice, ChR2-Amb/RVLM n=8 mice).

**b** Schematic of injection of AAV-DIO-ChR2-mCherry into the NTS followed by optical fiber implantation above the Amb for activation of the NTS-Amb pathway (left); and intragastric pressure and EMG were recorded during retching in mice, and retching behavioral was recorded by two orthogonally positioned cameras.

**(c-e)** Average curve and Statistical plot of peak of IGP (c), EMG (d), and opening angle (e) when optogenetic activation of the NTS-Amb pathway induced retching-like behavior.

**f** Quantitative analysis retching like behavior in mice of light activation NTS Calb1+ neurons following injection of CNO to inhibition of Amb neurons (n=8 mice).

Data are shown as mean± s.e.m. (error bars). Statistical analyses in c, d, e, f were performed using two-way ANOVA (***P<0.001).

**Supplementary Figure S9. Cereulide induced retching behavior.**

**a** Total number of retches for different doses of cereulide induced retching within 3 h in mice.

**b** Example micrograph and magnified fields from the NTS of WT mice induced retching behavior with cereulide showing colocalization of c-Fos^+^ with the Calb1^+^ neurons (Left); And quantitative analysis of the proportion of co-localized neurons in Calb1^+^ and c-Fos^+^ neurons scale bar=0.2 mm.

Data are shown as mean± s.e.m. (error bars). Statistical analysis in b was performed using two-sided Student t-tests (****P < 0.0001).

**Supplementary Figure S10. Classification of JNG neuronal subtypes by smart seq2 single-cell sequencing.**

**a** Schematic illustration of the methodology used in this study.

**b** Heatmap showing the expression profiles of differential gene in three different types of JNG neurons.

**Supplementary Figure S11. Retrograde tracing of Calb1^+^ NTS neurons with RV.**

**a** Example coronal section showing that the viral injection center, as indicated by the co-expression of EGFP and DsRed, was localized in the NTS.

**b** Eleven micrographs showing the projection fields of the NTS Calb1^+^ neurons. MOs6: Secondary motor area, layer 6; M1: primary motor cortex; M2: secondary motor cortex; S1: primary somatosensory cortex; S2: secondary somatosensory cortex; BST: Bed nuclei of the stria terminalis; AID: Agranular insular area, dorsal part; PVN: Paraventricular hypothalamic nucleus; CeA: Central amygdalar nucleus; BLA: Basolateral amygdalar nucleus; PSTN: Parasubthalamic nucleus; MVeMC: medial vestibular nucleus, magnocellular part; MVePC: medial vestibular nucleus, parvicellular; DPGi: dorsal paragigantocellular nucleus; IRt: intermediate reticular nucleus; FN: fastigial nucleus; MdV: medullary reticular nucleus, ventral part; LPGi: lateral paragigantocellular nucleus; Pr: prepositus nucleus; NTS: Nucleus of the

solitary tract.

**Supplementary Figure S12.** **JNG neurons single-cell RNA-seq data analysis.**

**a** Schematic illustration for preparation of JNG single-cell RNA.

**b** t-SNE visualization of DsRed^+^ cells colored by cluster identity.

**c** Dot plot showing the expression pattern of representative marker genes in all sub-cluster.

**d** t-SNE visualization of the expression patterns of cell type-specific marker genes; Grey to red indicates low to high expression levels, respectively.

**Supplementary Figure S13.** **Three types JNG neurons single-cell RNA sequence data analysis.**

**a** Dot plot showing DsRed expression across JNG sensory neuronal clusters (top); box plot showing the modulescores of type III neurons across JNG clusters using the DEGs of type III neurons (bottom).

**b** Heatmap of top marker genes of cereulide activated-JNG Type III neurons.

**Supplementary Figure S14. Summary diagram of the neural circuits involved in toxin-induced retching-like behavior.**

Abstract diagram showing a peripheral-to-central neural circuit mediates toxin-induced retching-like behavior in mice. In brief, when mice ingest contaminated food *(e.g.*, *B. cereus*), it is transmitting toxin-related signals via vagal ganglion neurons to the Calb1^+^ neurons in the NTS. Calb1^+^ neurons drive retching-like behavior (vomiting action) via sending projections to the nucleus ambiguus (Amb)/rostral ventrolateral medulla (RVLM) and then to the diaphragm; Calb1^+^ neurons drive nausea (vomiting memory, conditioned flavor avoidance) via sending projections to external lateral subdivision of the parabrachial nucleus (PBNel) and further to CeA.
