# Supplementary material for: Brain circuits for retching-like behavior
Source: Natl Sci Rev. 2023 Sep 27;11(1):nwad256. doi: 10.1093/nsr/nwad256 (PMC10824557; doi:10.1093/nsr/nwad256)
Supplement: nwad256_Supplemental_Files [file nwad256_supplemental_files.zip › Supplementary methods.docx]

**Supplementary information**

**Supplementary data of materials and methods**

Animals

The *Calb1*-2A-Cre, *Tac1*-ires-Cre, *Dbh*-2A-Flp, *Etv1*-CreER, *vGlut2*-ires-Cre, *GAD2*-ires-Cre, *Chat*-Cre and *FosCreER* (Fos-2A-iCreER) mouse lines were imported from the Jackson Laboratory (JAX Mice and Services). Mice were housed at room temperature (23 ± 1 °C) with a stable humidity (50 ± 5 %) and free access to food/water on a 12 h/12 h light/dark cycle. Mice were housed in groups (3–5 animals per cage) before they were separated 3 days before virus injection. After virus injection, each mouse was housed in one cage for 2 weeks before subsequent experiments. Because we did not observe statistic difference between male and female mice for *B. cereus* induced retching-like behavior (Supplementary information, Fig. 1a), almost the same amount of male and female mice was used in each experiments including control and test groups. All experimental procedures were conducted following protocols approved by the Administrative Panel on Laboratory Animal Care at the Guangzhou laboratory (Guangzhou, China).

AAV vectors

We used three AAV serotypes (AAV2/9, AAV2/8, AAV2-retro) in this study. The AAVs used in this study are listed in Supplementary Table S1. The viral particles were purchased from Shanghai Taitool Bioscience Inc. and Brain VTA Inc. The titers of viral vectors were initially in the range of 0.8-1.5 × 10^13^ particles/ml. The final titer used for AAV injection after dilution with PBS is 2 × 10^12^ viral particles/ml.

Stereotaxic viral injection

Mice were anesthetized using tribromoethanol (125-250 mg/kg) with intraperitoneal injection. Mice were placed on a stereotaxic frame (RWD Life Science, China) and received standard surgery that exposed the brain surface above the NTS, PBNel, Amb or CeA. Coordinates used for NTS injection were as follows: bregma −7.48 mm, lateral + 0.25 mm and dura −3.40 mm. Coordinates used for PBNel injection were as follows: bregma -5.20 mm, lateral ± 1.35 mm and dura −2.85 mm. Coordinates used for Amb/RVLM injection were as follows: bregma −6.85 mm, lateral ± 1.55 mm, dura -4.35 mm and 4° angle from the lateral to medial. Coordinates used for CeA injection were as follows: bregma −1.10 mm, lateral ± 2.55 mm, dura -3.95 mm. The AAVs were stereotaxically injected using a glass pipette connected to a Nanoliter Injector 201 (World Precision Instruments) at a slow flow rate of 0.15 μl/min to avoid potential damage to local brain tissue. The pipette was withdrawn at least 20 min after viral injection.

Optic fiber implantation

Thirty minutes after AAV injections, a ceramic ferrule with an optic fiber (optogenetics: 200 μm in diameter, N.A. 0.22; fiber photometry: 230 μm in diameter, N.A. 0.37) was implanted with the fiber tip on top of the NTS (bregma -7.48 mm, lateral -0.25 mm, and dura -3.15 mm), PBNel (bregma -5.20mm, lateral ±1.35 mm, and dura -2.60mm), Amb (bregma -6.85 mm, lateral ±1.55 mm, dura -4.10 mm, 4 angle from the lateral to medial). The ferrule was then secured on the skull with dental cement. After implantation, the skin was sutured and antibiotics were applied to the surgical wound. The optogenetic and fiber photometry experiments were conducted at least 3 weeks after optic fiber implantation. For optogenetic manipulations, the output of the laser was measured and adjusted to 5 mW, 10 mW and 20 mW before each experiment. The pulse onset, duration and frequency of light stimulation were controlled by a programmable pulse generator attached to the laser system. After AAV injection and fiber implantation, the mice were housed individually for 3 weeks before the behavioral tests.

Fiber photometry

Calcium signal was recorded by a commercialized fiber photometry system (Thinker Tech Nanjing Biotech CO., Ltd). AAV-DIO-GCaMP6s or AAV-DIO-EGFP was stereotaxically injected into the NTS of *Calb1*-2A-Cre mice. After the virus injection, the optical fiber (diameter, 230 μm, NA = 0.37; Fiblaser Technology Co., Ltd) was planted above NTS. A fiber photometry system (ThinkerTech) was used for recording GCaMP signals from genetically identified neurons [^1^](#_ENREF_1).To induce fluorescence signals, a laser beam from a laser tube (488 nm) was reflected by a dichroic mirror, focused by a 10× len (NA = 0.3) and then coupled to an optical commutator. A 3-m optical fiber (200 mm O.D., NA = 0.37) guided the light between the commutator and the implanted optical fiber. To minimize photobleaching, the power intensity at the fiber tip was adjusted to 0.02 mW. The GCaMp6s [^2^](#_ENREF_2) and the acetylcholine indicator fluorescence was band-pass filtered (MF525-39, Thorlabs) and collected by a photomultiplier tube (R3896, Hamamatsu). An amplifier (C7319, Hamamatsu) was used to convert the photomultiplier tube current output to voltage signals, which was further filtered through a low-pass filter (40 Hz cut-off; Brownlee 440). The analog voltage signals were digitalized at 100 Hz and recorded by a Power 1401 digitizer and Spike2 software (CED, Cambridge, UK). Two weeks after AAV injection, fiber photometry was used to record GCaMP signals. A flashing light-emitting diode triggered by a 1-s square-wave pulse was simultaneously recorded to synchronize the video and GCaMP signals. For recordings from freely moving mice, mice with optical fibers connected to the fiber photometry system freely explored the arena for 10 min. *B. cereus* was administered to mice by gavage to induce retching behavior, while the GCaMP signals and retching behaviors were simultaneously recorded. As a control, *B. cereus* was administered to mice injected with a control virus (AAV-DIO-EGFP). Normalized GCaMP fluorescence (ΔF/F) was used to measure the GCaMP signal and align it with the starting point of the freely explored. After the experiments, the optical fiber tip sites were histologically examined in each mouse.

Slice physiology

Slice physiological recording was performed according to a previously published protocol [^3^](#_ENREF_3). Brain slices containing the NTS, PBNel, or Amb were prepared from adult mice anesthetized with isoflurane before decapitation. Brains were rapidly removed and placed in ice-cold oxygenated (95% O_2_ and 5% CO_2_) cutting solution (CS: 228 mM sucrose, 11 mM glucose, 26 mM NaHCO_3_, 1 mM NaH_2_PO_4_, 2.5 mM KCl, 7 mM MgSO_4_, and 0.5 mM CaCl_2_). Coronal brain slices (250 μm) were cut using a vibratome (VT 1200S, Leica Microsystems, Wetzlar, Germany). The slices were incubated at 28 °C in oxygenated artificial cerebrospinal fluid (ACSF: 119 mM NaCl, 2.5 mM KCl, 1 mM NaH_2_PO_4_, 1.3 mM MgSO_4_, 26 mM NaHCO_3_, 10 mM glucose, and 2.5 mM CaCl_2_) for 30 min, and were then kept at room temperature under the same conditions for 1 h before transfer to the recording chamber at room temperature. The ACSF was perfused at 1ml per min. The NTS slices were visualized with a ×40 Olympus water immersion lens, differential interference contrast (DIC) optics (Olympus Inc., Japan), and a CCD camera (QImaging Rolera-XR, BC, Canada). Patch pipettes were pulled from borosilicate glass capillary tubes (Cat #64-0793, Warner Instruments, Hamden, CT, USA) using a PC-10 pipette puller (Narishige Inc., Tokyo, Japan). For recording of action potentials (current clamp), pipettes were filled with solution (in mM: 135 K-methanesulfonate, 10 HEPES, 1 EGTA, 1 Na-GTP, 4 Mg-ATP, pH 7.4). For recording of postsynaptic currents (voltage clamp), pipettes were filled with solution (in mM, 135 CsCl_2_, 10 HEPES, 1 EGTA, 1 Na-GTP, 4 Mg-ATP, pH 7.4). The resistance of pipettes varied between 3.0 and 3.5 MΩ. The current and voltage signals were recorded with MultiClamp 700B and Clampex 10 data acquisition software (Molecular Devices). After establishment of the whole-cell configuration and equilibration of the intracellular pipette solution with the cytoplasm, series resistance was compensated to 10-15 MΩ. Recordings with series resistances of >15 MΩ were rejected. An optic fiber (200 μm in diameter) was positioned above the brain slices, with laser intensity adjusted to 20 mW. Light-evoked action potentials from ChR2-mCherry^+^ neurons were triggered by light-pulse train (473 nm, 1ms, 10 Hz, 20 mW) synchronized with Clampex 10 data acquisition software (Molecular Devices). Light-evoked synaptic currents from ChR2-mCherry-negative NTS neurons were triggered by single light pulses (1 ms) in the presence of 4-AP (20 μM) and TTX (1 μM). D-AP5 (50 μM)/CNQX (20 μM) or picrotoxin (PTX, 50 μM) or biocuculin (BIC, 20 μM) were perfused with ACSF to examine the neurotransmitter type used by ChR2-mCherry-expressing neurons.

JNG neuron culture

Primary cultures of JNG neurons were prepared from mice as previous studies described [^4^](#_ENREF_4). In brief, mice were deep anesthetized and then the ganglia were rapidly removed and placed into the D-Hanks solution (Gibco). Ganglia samples were digested at 37 °C with collagenase type 2 and Dispase II (2 mg/mL and 7.5 mg/mL) for 30 min. They were subsequently suspended in 10 mL DMEM(Gibco) plus 10% bovine calf serum (Gibco) to stop digestion. Ganglia were then dissociated into a suspension of individual cells and plated onto 13 mm glass coverslips pre-coated with 100 g/mL poly-D-lysine (BD Biosciences). Cells were incubated at 37 °C with a 5% CO2 and 95% O2. WT mice JNG neurons were used for patch clamp recording within 24 h.

JNG neurons action potentials recording

Whole-cell mode patch clamp recordings were performed at a room temperature of 22-24 ◦C. Coverslips with cultured JNG neurons were placed in a 0.5 mL microchamber. The bath solution contained (in mM) 145 NaCl, 5 KCl, 2 MgCl_2_, 2 CaCl_2_, 10 HEPES and 10 Glucose with an osmolarity 320 mOsm. The pH was adjusted to 7.35 with NaOH. The internal recording solution contained (in mM) 135 K-methanesulfonate, 10 HEPES, 1 EGTA, 1 Na-GTP, 4 Mg-ATP, pH was adjusted to 7.35 with KOH and osmolarity was adjusted with sucrose to 320 mOsm. In the current-clamp mode, the excitability of JNG neurons before and after cereulide (0.5 ng/ml) administration was recorded. Current injections in steps of 20 pA, starting at -20 pA and increasing up to 200 pA with a step duration of 300 ms were applied to characterize the neuronal discharge behavior.

After recording, the JNG neurons were aspirated (under a microscope) into a patch pipette using a conventional patch-clamp setup with negatively pressurised pipette holder. The electrode tip was then quickly broken into a 0.2-mL PCR tube containing 4 μL lysis buffer for subsequent experiments.

Histological procedures

The procedure of the immunohistochemical was performed following a previously published protocol [^5^](#_ENREF_5). Mice were anesthetized with tribromoethanol (125-250 mg/kg) and sequentially perfused with saline and PBS containing 4% paraformaldehyde. Brains were removed and incubated in PBS containing 30% sucrose until they sank to the bottom. Post-fixation of the brain was avoided to optimize immunohistochemistry of GABA and glutamate. Cryostat sections (40 μm) containing the NTS, PBNel, Amb or CeA were collected, incubated 2h with blocking solution (PBS containing 10% goat serum and 0.7% Triton X-100), and then treated with primary antibodies diluted with blocking solution for overnight at 4 ℃. Primary antibodies used for immunohistochemistry are provided in Supplementary Table S1. Primary antibodies were washed three times with washing buffer (PBS containing 0.7% Triton X-100) before incubation with secondary antibodies (tagged with Cy2, Cy3 or Cy5; dilution 1:500;) for 2 h at room temperature. Sections were then washed three times with washing buffer, stained with DAPI, and then washed with PBS. Sections were transferred onto Super Frost slides and mounted under glass coverslips with mounting media. Sections were imaged using a Leica Aperio versa 8 microscope (10 objective lens) or a Zeiss 900 laser scanning confocal microscope (20 and 60 oil-immersion objective lens). Samples were excited by 488, 546 or 633 nm lasers in sequential acquisition mode to avoid signal leakage. Confocal images were analyzed using the software Image J.

Preparation of *B. cereus*

For preparation the strains *B. cereus* (it was kindly provided by professor Zhu Kui, Beijing Advanced Innovation Center for Food Nutrition and Human Health, College of Veterinary Medicine, China Agricultural University) preserved in glycerin were streaked in plates, cultured at 37 ℃ overnight, and the single colony was selected. Inoculate the activated strains into 100 mL medium overnight until the OD value is between 0.4-0.5, *B. cereus* were collected and centrifuged at 4000 rpm for 10min, and were stored at 4 ℃.

Preparation of acute gastritis model

The experimental mice were habituated individually for one day, then fasted and water deprived for 10 hours before each mouse was intragastric administrated with 2% aspirin (Bayer) and 0.6 M HCL solution, 10 μL/g.

*B. cereus* induced retching behavior

The stored strains were resuspended with 300 μL medium and returned to room temperature. The acute gastritis mice were continually fasted and water deprived until 24 h, and then adapted in a transparent acrylic chamber (20 cm x 20 cm, square open field) for 15 min. Mice were intragastric administrated *B. cereus* or vehicle with 10 μL/g once an hour for a total of 3 hours. One hour after administration of *B. cereus*, the mice began to show retching behaviors with their mouths opening. However, mice who were intragastric administrated vehicle did not exhibit retching behaviors. The retching behaviors were recorded by two orthogonally positioned cameras (50 frames/s; Point Grey Research, Canada).

Cell-counting strategies

For counting cells in the JNG neurons, we collected 15-μm coronal sections for each mouse. Five sections evenly spaced by 75 μm were sampled for immunohistochemistry to label cells positive for different markers. We acquired confocal images (ZEISS LSM900 microscope, ×10 objective) within intermediate and deep layers of the JNG followed by cell counting with Image J software. We counted the numbers of NeuN^+^ neurons and calculated the average in the neuronal population retrogradely labeled by RV-mCherry.

For counting cells in the NTS, we collected coronal sections (40 μm) from bregma -7.32 to bregma-7.52 for each brain. Three sections spaced by 200 μm were sampled for immunohistochemistry to label cells positive for different markers. We calculated the average in NTS neurons with neuronal population labeled by different molecular tools (AAV-DIO-retro-EGFP in Amb/RVLM; AAV-DIO-retro-mCherry in PBNel).

For counting cells in the whole brain, we collected coronal sections (40 μm) for each mouse. One sheet every 200 μm was taken for immunohistochemistry to label positive cells. We retrogradely labeled these nucleus (CeA: central amygdaloid nucleus, PSTH: parasubthalamic nucleus and some cortexs: M1; M2; A1; Au1; AuV; D1; G1; S1; S2) We acquired micrographs (×10 objective) of these nucleuses and calculated the average of RV-mCherry^+^ neurons.

FosTRAP strategies

AAV-hsyn-DIO-hM3D-mCherry was unilaterally injected into the NTS of *FosCreER* (Fos-2A-iCreER) mice on day 1. 4-Hydroxytamoxifen was dissolved in corn oil (20 mg/mL). On day 7, acute gastritis model was established. *B. cereus* was intragastric administration to induced retching (retching-TRAP) or medium (control: no-retching-TRAP). After 2 times of *B. cereus* or medium administration (1h / time), 4-hydroxytamoxifen (12.5 mg/kg) was intraperitoneally injected into the *FosCreER* mice. Then *B. cereus* or medium was administration once more. On Day 21 (2 weeks after retching-TRAP), the acute gastritis model was established again and *B. cereus* or medium was administrated to the mice, followed by perfusion. Coronal sections were collected for immunohistochemistry for mCherry and c-Fos.

Conditioned flavor avoidance behavior

The procedure of the CFA was modified from a previously published method [^6^](#_ENREF_6). AAV-hEF1a-DIO-hChR2-mCherry was stereotaxically injected into the NTS of *Calb1*-2A-Cre mice. After the virus injection, the optical fiber was planted above Amb (Amb group) or PBNel (PBN group). Two weeks after AAV injection, mice were transferred to a homecage containing two water bottles for three days. Both water bottles contained unflavored water and mice can drink water freely. On day four, both water bottles were filled with either grape-flavored or orange-flavored water for three days. During those three days, the grape or orange-flavored water intake of the mice was weighed daily. On day seven, mice were fasted water for 12 h. On day eight (conditioning day), both water bottles were filled with either grape-flavored or orange-flavored water and connected with 1401 digitizer. Grape-flavored bottle also connected with laser light source, when mice drinking grape bottle, light stimulation was triggered. After 2 hours of testing, the grape or orange-flavored water intake of the mice was weighed. On day nine (recover day), both water bottles contained unflavored water. On day ten (testing day), one water bottle contained orange-flavored water, while the other contained grape-flavored water on a randomized arena side, and consumption from each water bottle as an evaluation indicator.

Cell-type-specific RV tracing

The modified RV based three-virus system was used for retrograde tracing. All the viruses included AAV2/9-EF1a-DIO-EGFP-2A-TVA (5 x 10^12^ viral particles/mL), AAV2/9-EF1a-DIO-RG (5 x 10^12^ viral particles/mL), and EnvA-pseudotyped, glycoprotein (RG)-deleted and DsRed-expressing RV (RV-EnvA-DsRed, RV) (5.0 x 10^8^ viral particles/mL). For mapping the whole-brain inputs to Calb1^+^ NTS neurons, a mixture of AAV2/9-EF1a-DIO-EGFP-2A-TVA and AAV2/9-EF1a-DIO-RG (1:1, 150 nL) was stereotaxically injected into the NTS of *Calb1*-2A-Cre mice unilaterally. Two weeks after AAV helper injection, RV-EnvA-DsRed (150 nL) was injected into the same location in the NTS of *Calb1*-2A-Cre mice in a biosafety level-2 lab facility. Starter neurons were characterized by the coexpression of DsRed and EGFP, which were restricted in the NTS. For the cell-type-specific Retrograde tracing of CeA-PBNel-Calb1^+^ NTS neurons, RV-helper (AAV2/9-EF1a-DIO-EGFP-2A-TVA and AAV2/9-EF1a-DIO-RG) was stereotaxically injected into the PBNel of *vGlut2*-Cre mice unilaterally. Two weeks after AAV helper injection, RV-EnvA-DsRed (200 nL) was injected into in the CeA of the same mice. One week after injection of RV, mice were perfused with saline followed by 4% paraformaldehyde (PFA) in PBS. The brain and NG were post-fixed in 4%PFA for 8 hours and then incubated in PBS containing 30% sucrose until they sank to the bottom. Coronal brain sections at 40 μm and NG sections at 10 μm in thickness were prepared using a cryostat (Leica CM1900). All sections were collected and stained with DAPI or other antibodies. The sections were imaged with an Aperio versa 8 microscope (10 x objective lens) and analyzed with ImageJ. For quantifications of subregions, boundaries were based on the Allen Institute’s reference atlas. We selectively analyzed the retrogradely labeled dense areas. The factional distribution of total cells labeled by RV was measured.

Cell-type-specific anterograde tracing

For cell-type-specific anterograde tracing of Calb1^+^ NTS neurons, AAV-DIO-H2B-EGFP-mRuby or AAV-DIO-EGFP-syb2 was stereotaxically injected into the NTS of *Calb1*-2A-Cre mice. The mice were then maintained in a cage individually. Three weeks after viral injection, mice were perfused with saline followed by 4% PFA in PBS. The brains were post-fixed in 4% PFA for 8 hours and then incubated in PBS containing 30% sucrose until they sank to the bottom. Coronal brain sections at 40 μm thickness were prepared using a cryostat (Leica CM1900). All coronal sections were collected and stained with primary antibody against mCherry and DAPI. The coronal brain sections were imaged with an Aperio versa 8 microscope (10 x objective lens).

Preparation of behavioral tests

After AAV injection and fiber implantation, the mice were housed individually for 3 weeks before the behavioral tests. They were handled daily by the experimenters for at least 3 days before the behavioral tests. On the day of behavioral test, we moved the mouse cages to the testing room and habituated the mice to the room conditions for 3 h before the experiments started. The apparatus was cleaned with ethanol (20%) to eliminate odor cues from other mice. The behavioral tests were performed in the same circadian period (1 PM–7 PM). All behaviors were scored by the experimenters who were blind to the animal treatments.

Measuring chemogenetically evoked retching behavior

On the day of experiment, the test mouse was allowed to explore an area (20cm x 20cm, square open field) for 15 minutes. Then the test mouse was intraperitoneally injected with CNO (1mg/kg) or saline (as a control) and returned to the area, allowing the CNO to gradually activate the NTS neurons that express hM3Dq-mCherry. The behavior of the test mouse was recorded by two orthogonally positioned cameras (50 frames/s; Point Grey Research, Canada). The retching behavior was identified by visual observation of the mouth opening response of mice. The chemogenetically-evoked retching behavior was quantified by measuring the numbers of mouth opening in mice during the 1 hours after the injection of CNO. Finally, the effectiveness of CNO to evoke action potential firing from NTS neurons was validated with slice physiology.

Measuring photostimulation-evoked retching behavior

On the day of experiment, the test mouse with optic fiber implantation above the NTS was allowed to explore an area (20cm x 20cm, square open field) for 15 minutes. Then light stimulation was applied to optogenetically activate NTS neurons. Laser power and frequency of light pulses were adjusted to measure the dependence of retching behavior on different parameters of light stimulation. The behavior of test mouse was recorded with two orthogonally positioned cameras (50 frames/s; Point Grey Research, Canada). The retching behavior was identified by visual observation of the mouth opening response of mice. The light-evoked retching behavior was quantified by measuring the number of mouth openings in mice that was evoked by light stimulation. Six light stimulation protocols [(10 Hz, 5 mW, 10 ms, 5 s), (10 Hz, 10 mW, 10 ms, 5 s), (10 Hz, 20 mW, 10 ms, 5 s), (5 Hz, 10 mW, 10 ms, 5 s), (10 Hz, 10 mW, 10 ms, 5 s), (20Hz, 10 mW, 10 ms, 5 s)] were used to test the dependence of retching behavior in mice on laser power and light stimulation frequency.

Measuring retching behavior after chemogenetic inhibition of NTS neurons

AAV-hsyn-DIO-hM4D-mCherry was unilaterally injected into the NTS of *Calb1*-2A-Cre mice. Two weeks after AAV injection, acute gastritis model was established. The next day, test mouse was allowed to explore an area (20cm x 20cm, square open field) for 15 minutes. *B. cereus* was intragastric administrated to induced retching. Then the test mouse was intraperitoneally injected with CNO (1mg/kg) or saline (as a control), followed by returning to the area, allowing the CNO to gradually inhibit the NTS neurons that express hM4Di-mCherry. After 30 minutes, the test mouse was subject to the test of *B. cereus*-induced retching behavior and continuous recording for 1 hour. The behavior of the test mouse was recorded by two orthogonally positioned cameras (50 frames/s; Point Grey Research, Canada). The effectiveness of CNO to suppress action potential firing from NTS neurons was validated with slice physiology.

Measuring retching behavior after inhibition of NTS-Amb/RVLM pathway

AAV-hsyn-hM4D-mCherry was bilaterally injected into the Amb/RVLM of *Calb1*-2A-Cre mice. Meanwhile, AAV-DIO-ChR2-EYFP was unilaterally injected into the NTS. After the virus injection, the optical fiber was planted above NTS. After AAV injection and fiber implantation, the mice were housed individually for 3 weeks before the behavioral tests. On the day of experiment, the test mouse was allowed to explore an area (20cm x 20cm, square open field) for 15 minutes. Then light stimulation was applied to optogenetically activate NTS neurons. The behavior of test mouse was recorded with two orthogonally positioned cameras (50 frames/s; Point Grey Research, Canada). The retching behavior was identified by visual observation of the mouth opening response of mice. The light-evoked retching behavior was quantified by measuring the number of mouth openings in mice that was evoked by light stimulation. Two light stimulation protocols [(10 Hz, 5 mW, 10 ms, 5 s), (10 Hz, 20 mW, 10 ms, 5 s)] were used to test the dependence of retching behavior in mice on laser power and light stimulation frequency. Then the test mouse was intraperitoneally injected with CNO (1mg/kg), followed by returning to the area, allowing the CNO to gradually inhibit the Amb/RVLM neurons that express hM4D-mCherry. After 20 minutes, we used the same light stimulation protocols to activate NTS neurons and recorded retching behavior in mice.

Measuring vomitus of retching mice

AAV-DIO-ChR2-mCherry or AAV-DIO-mCherry were stereotaxically injected into the NTS of Calb1-2A-Cre mice as test or control groups, respectively. After virus injection, the optical fiber was implanted above the RVL/Amb. After AAV injection and fiber implantation, the mice were housed individually for 3 weeks. On the day of experiment, the test mouse was allowed to explore an area (20cm x 20cm, square open field) for 15 minutes. Mice were slowly given dragon fruit juice (1 mL/10 g, approximately 2.5 mL/each mouse) by gavage. And then the mice were returned to the cage and had a rest for 10min. Before laser stimulation, the residual dragon fruit juice around mice mouth was cleaned up, and then a laser stimulation protocol (10 Hz, 10 mW, 10 ms, 20 s) was given to induce intense retching behaviors in the mice. The strong light-activated evoked mice to vomit some of the dragon fruit juice. Red dragon fruit juice was more obvious than other foods. The weight of the vomitus can be calculated by taking a clean paper and weighing it, then wiping the vomitus with the paper and weighing it again.

Measuring gastric pressure and EMG

After the mice were anesthetized with tribromoethanol (125 - 250 mg/kg), the abdomen was cut open to expose the stomach and diaphragm. A latex balloon (VK, 73-3478) connected to PE-160 tubing (external diameter 0.5mm) was inserted into the stomach. A double-strand tungsten microelectrode (A-M Systems, 795500) with the insulating layer removed from the tip (~0.2 mm) was inserted into the left costal region of the left diaphragm via a [syringe](javascript:;) [needle](javascript:;) as a guide. The tubing and microelectrode were passed subcutaneously and exited at the top of the scalp near the connector that used for EMG and gastric pressure recordings. The EMG signals were recorded using a Microelectrode AC Amplifier Model 1800 (A-M Systems), filtered (100-5000 Hz EMG recordings) and digitized at 5000 Hz using Spike2 software (Cambridge Electronic Design). Gastric pressure was recorded with the barometer (Benetech, GM510).

Surgical procedures of PRV injection

Surgical procedures are similar to EMG surgery. After adequate exposure of the diaphragm, a total volume of 3 μL PRV virus (2 x 10^9^ PFU/mL) was injected at three sites (1 μL for each site) across the costal region of the left diaphragm using a 2.5 μL Hamilton syringe with a bevelled 34 gauge needle. 5 days after injection of PRV, mice were perfused with saline followed by 4% PFA in PBS.

Single-nuclei isolation

Three WT adult mice were anesthetized with isoflurane and brains were quickly removed and placed in artificial cerebral spinal fluid (ACSF, 4 ℃) solution (ACSF: 119 mM NaCl, 2.5 mM KCl, 1 mM NaH_2_PO_4_, 1.3 mM MgSO_4_, 26 mM NaHCO_3_, 10 mM glucose, and 2.5 mM CaCl_2_) bubbled with 95% O_2_ and5% CO_2_. Coronal slices at 250 μm were made using a vibratome (VT 1200S, Leica Microsystems, Wetzlar, Germany) in ACSF. The NTS was visualized by microscopy and harvested based on anatomical landmarks. Isolated NTS tissue were placed in a tube with RNAlater (Invitrogen, AM7020), samples were then flash-frozen in liquid nitrogen, and stored at -80 ℃ for later use. Nuclei Isolation Kit (Sigma-Aldrich, NUC201-1KT) was used to isolate single nuclei, and single nuclei were resuspended in cold PBS (with 1% BSA and 0.2 U/μL RNase inhibitor) at a concentration of 800 ~ 1,000 nuclei/μl for downstream applications.

10X Single-nucleus RNA-sequencing (snRNA-seq) library preparation and sequencing

snRNA-seq libraries were generated using Chromium™ Next GEM Single Cell 3’ GEM Library & Gel Bead Kit v3.1 (10X Genomics, Cat# 1000121) according to the manufacturer’s protocol. Sequencing was performed on the Illumina NovaSeq 6000 v1.5 platform with a sequencing depth of at least 100,000 reads per cell.

Preparation of JNG single-cell suspensions

A mixture of AAV2/9-EF1a-DIO-EGFP-2A-TVA and AAV2/9-EF1a-DIO-RG (1:1, 150 nL) was stereotaxically injected into the NTS of *Calb1*-2A-Cre mice unilaterally. Two weeks after AAV helper injection, RV-EnvA-DsRed (150 nL) was injected into the same location in the NTS of *Calb1*-2A-Cre mice in a biosafety level-2 lab facility. Seven days later, 10 *Calb1*-2A-Cre mice that had been injected with RV virus were anesthetized with isoflurane. JNG were quickly removed and placed in hank’s solution. The dissection time of each mouse was controlled within 10 min. After the ganglia were removed the dissociation protocol was started without delay as previously published protocol [^7^](#_ENREF_7). Briefly, the isolated ganglia were transferred into a 3 cm plastic dish with 2 mL digestion solution. The digestion solution consisted of 400 μL TrypLE Express (Life Technologies; cat #12605-010), 1800 μL Papain solution (Worthington Biochemical; cat #LK003178; 25U/ml in CS), 100 μL DNase I (Worthington Biochemical; cat #LK003172; 1mM in CS) and 200 μL Collagenase/Dispase (Roche; 20 mg/mL in CS). Every 20 min tissue was triturated using glass Pasteur pipettes (pretreated with 0.5% BSA solution) with decreasing diameter. After of approximately 60 min the dissociation was complete and single cells became visible at the bottom of the plastic dish. The solution was filtered using a 40 mm cell strainer (FALCON) and collected in a 15 mL plastic tube containing 3 mL CS and centrifuged at 100 g for 5 min at 4 ℃. The supernatant was removed and the pellet resuspended in 0.5 mL ASCF and 0.5 mL complete Neurobasal medium (Neurobasal-A supplemented with L-Glutamine, B27 (all GIBCO) and Penicillin/Steptamycin (Sigma)). The cell suspension was carefully transferred with a Pasteur pipette and layered on top of an Optiprep gradient: 100 μL Optiprep Density Solution (Sigma) in 450 μL ACSF and 450 μL complete Neurobasal; The gradient was centrifuged at 70 g for 10 min at 4 ℃, the supernatant removed and resuspended with cold PBS + 0.04% BSA + 0.2 U/μL RNase inhibitor at a concentration of 800 ~ 1,000 nuclei/μL for downstream applications.

JNG neuron single-cell library preparation

After electrophysiological recording, the JNG neurons were aspirated (under a microscope) into a patch pipette using a conventional patch-clamp setup with negatively pressured pipette holder. The electrode tip was then quickly broken into a 0.2-mL PCR tube containing 4 μL lysis buffer (contain: oligo-dT primer, RNase inhibitor, 1% Triton X-100, dNTP mix) for subsequent experiments.

Reverse transcription and cDNA amplification were performed following SMART-seq2 protocol [^8^](#_ENREF_8). After cDNA amplification, 25 μL of the sample were subjected to cDNA purification on AMPure XP beads. 5 ng of each purified cDNAds were used to construct the sequencing library using TruePrep® DNA Library Prep Kit V2 for Illumina（Vazyme, Cat#TD502） with fragments over 300-bp length from each neuron.

NTS single cell snRNA-seq data processing

Unique molecular identifier (UMI) counts were generated for each barcode using the Cell Ranger (version 5.0.1) [^9^](#_ENREF_9) count utility. with --include-introns parameter. Count data were processed with the Seurat package (version 4.1.0) [^10^](#_ENREF_10). For quality control, the doublets identified by DoubletFinder (version 2.0.3) [^11^](#_ENREF_11) were filtered cells that satisfy the following conditions nFeature_RNA > 500 and < 6,000, nCount_RNA >1,000 and < 20,000, percent.mt < 1 were kept. After quality control, cells from different samples were then merged by *merge* function, and gene expression counts were normalized with *NormalizedData* function, were scaled and centred using *ScaleData* function. Batch-effect correction was performed by Harmony (version 0.1.0) [^12^](#_ENREF_12) after principal component analysis. And then a shared-nearest neighbour graph based on Harmony embeddings was built. The SLM (smart local moving algorithm) algorithm were implemented through the *FindClusters* function to cluster the cells. Clusters were visualized using Uniform Manifold Approximation and Projection (UMAP). Cluster-specific marker genes were identified by *FindAllMarkers* function. The cell type of the individual clusters was assigned using canonical marker genes. In detailed, clusters were annotated as the following groups: excitatory neurons (*Slc17a7*), inhibitory neurons (*Gad1*, *Gad2*), oligodendrocytes (*Mbp*, *Mog*), oligodendrocyte precursor cells (*Pdgfra*), astrocyte (*Aqp4*, *Gfap*, *Slc15a2*), endothelial cells (*Abcc9*, *Pecam1*, *Cdh5*), microglia (*Cx3cr1*, *Csf1r*, *Cd53*), and ependymal (*Spef2*). And then Neuron cells were reclustered and identified sixclusters using 0.1 resulution. FindAllMarkers function was used to find cluster specific marker genes. Gene ontology (GO) enrichment analysis for the cluster marker genes using Metascape [^13^](#_ENREF_13).

JNG single cell scRNA-seq data processing

For JNG sample, RFP sequence was added to the mouse genome and Cellranger mkref was used to make genome reference. And count data were processed with the Seurat package. The doublets were also filtered by DoubletFinder. Cell and gene filtering was performed as follows: nFeature_RNA > 100 & nFeature_RNA < 5,000 & nCount_RNA > 500 & nCount_RNA < 50,000 & percent.mt < 5). The downstream analysis including data normalization, variable features identification, data scaling, dimension reduction, batch-effect correction, clustering, cluster marker genes identification followed the steps above. Clusters were visualized using the t-distributed Stochastic Neighbor Embedding (t-SNE). Clusters were annotated as the following groups: sensory neurons (*Phox2b*, *Slc17a6*, satellite glial cells (*Apoe*, *Plp1*), endothelial cells (*Peacm1*, *Cdh5*), erythrocyte (*Alas2*), fibroblast cells (*Lum, Pdgfra*), B cells (*Cd79a, Cd19*), T cells (*Cd3e, Cd3g*), NK cells (*Klrd1, Nkg7*) and antigen-presenting cells (*C1qa, Cd74*). And then sensory neuron cells were reclustered and identified 31 clusters using 0.6 resulution. *FindAllMarkers* function was used to find cluster specific marker genes.

JNG scRNA-seq data processing

The FASTQ files were quality controlled to cut adaptor and remove low quality reads using TrimGalore (https://github.com/FelixKrueger/TrimGalore).The clean data were mapped to the mouse genome (GRCm38) using HISAT2 (vesion 2.2.1) [^14^](#_ENREF_14) and the read count for each gene was calculated using featureCounts function. The count matrix was also processed with the Seurat package (v4.1.0) using 1000,000 as scale factor. *FindAllMarkers* function was used to identify differentially expressed genes between the control, inhibited and activated group. *AddModuleScore* function was used to calculate a cluster-level module activity score with nbin =10 for the 31 clusters of neurons from JNG based on the genes found above. Before scoring, the 31 clusters were psudobulk processed using the *Average Expression* function from Seurat.

Manufacturers of the instruments used in the article

| **Instrument name** | **Manufacturer** |
| --- | --- |
| optogenetic laser | RWD |
| Brain Stereotactic Injector | RWD |
| Isolated high power stimulator | A-M systems |
| Data Acquisition System | CED |
| Fiber photometry | ThinkerTech Nanjing Bioscience Inc |
| Axon Digidata 1550B Low Noise Data Acquisition System | Axon |
| [Axon Multiclamp 700B](https://biomed-labx.com/products/axon-multiclamp-700b/) | Axon |
| MPC-200 | Sutter instrument |

Intragastric administration for mice

With one hand, the mouse's tail was lifted onto a rough surface, while the other hand grasped the mouse and fixed the head, trunk and tail of the mouse so that the mouse's head and trunk were kept in a straight line and in a head-high-tail-low position. The end of the 11-gauge gavage tube was inserted through the corner of the mouse's mouth, and slowly inserted into the stomach along the oesophagus of the mouse, feeling no resistance during the insertion of the gavage tube. The drug was then slowly injected, and if the mouse did not respond, the remaining drug was rapidly injected. Finally, the tube was slowly removed and the mice were returned to the cage.

Statistical analysis

Data were presented as the Mean ± SEM and analyzed using GraphPad Prism 8. Statistical significance was evaluated using either a Student’s t-test or one-way analysis of variance (ANOVA) followed by Dunnett’s post hoc test for multiple groups or two-way ANOVA followed by Bonferroni’s multiple comparisons test. P < 0.05 was considered to indicate a statistically significant difference.

**References**

1 Li, Y. *et al.* Hypothalamic Circuits for Predation and Evasion. *Neuron* **97**, 911-924 e915 (2018).

2 Chen, T. W. *et al.* Ultrasensitive fluorescent proteins for imaging neuronal activity. *Nature* **499**, 295-300 (2013).

3 Liu, Z. *et al.* IGF1-Dependent Synaptic Plasticity of Mitral Cells in Olfactory Memory during Social Learning. *Neuron* **95**, 106-122 e105 (2017).

4 Jia, Z., Ikeda, R., Ling, J., Viatchenko-Karpinski, V. & Gu, J. G. Regulation of Piezo2 Mechanotransduction by Static Plasma Membrane Tension in Primary Afferent Neurons. *J Biol Chem* **291**, 9087-9104 (2016).

5 Shang, C. *et al.* A subcortical excitatory circuit for sensory-triggered predatory hunting in mice. *Nat Neurosci* **22**, 909-920 (2019).

6 Zhang, C. *et al.* Area Postrema Cell Types that Mediate Nausea-Associated Behaviors. *Neuron* **109**, 461-472.e465 (2021).

7 Kupari, J., Häring, M., Agirre, E., Castelo-Branco, G. & Ernfors, P. An Atlas of Vagal Sensory Neurons and Their Molecular Specialization.

8 Picelli, S. *et al.* Full-length RNA-seq from single cells using Smart-seq2.

9 Zheng, G. X. *et al.* Massively parallel digital transcriptional profiling of single cells. *Nature communications* **8**, 14049 (2017).

10 Hao, Y. *et al.* Integrated analysis of multimodal single-cell data. *Cell* **184**, 3573-3587.e3529 (2021).

11 McGinnis, C. S., Murrow, L. M. & Gartner, Z. J. DoubletFinder: Doublet Detection in Single-Cell RNA Sequencing Data Using Artificial Nearest Neighbors. *Cell Syst* **8**, 329-337 e324 (2019).

12 Korsunsky, I. *et al.* Fast, sensitive and accurate integration of single-cell data with Harmony. *Nature methods* **16**, 1289-1296 (2019).

13 Zhou, Y. *et al.* Metascape provides a biologist-oriented resource for the analysis of systems-level datasets. *Nat Commun* **10**, 1523 (2019).

14 Kim, D., Paggi, J. M., Park, C., Bennett, C. & Salzberg, S. L. Graph-based genome alignment and genotyping with HISAT2 and HISAT-genotype. *Nat Biotechnol* **37**, 907-915 (2019).
