# Supplementary material for: Brain circuits for retching-like behavior
Source: Natl Sci Rev. 2023 Sep 27;11(1):nwad256. doi: 10.1093/nsr/nwad256 (PMC10824557; doi:10.1093/nsr/nwad256)
Supplement: nwad256_Supplemental_Files [file nwad256_supplemental_files.zip › Supplementary Movie legends.docx]

**Supplementary information**

**Supplementary movie legends**

**Supplementary movie S1. Recording of *B. cereus*-induced retching**, related to Figure 1. Movie S1 shows *B. cereus*-induced retching of an example WT mouse recorded with high-speed camera (top) and gastric pressure and mouth opening angle were recorded during retching (bottom).

**Supplementary movie S2. Effects of chemogenetic activation of *B. cereus*-TRAPed NTS neurons**, related to Figure 1. Movie S2 shows that chemogenetic activation of *B. cereus*-TRAPed NTS neurons caused retching behavior in mice. Movie shows 1x and 0.2x speed.

**Supplementary movie S3. Effects of chemogenetic activation of Calb1^+^ NTS neurons**, related to Figure 3. Movie S3 shows chemogenetic activation of Calb1^+^ NTS neurons evoked intense retching behavior in an example mouse. The test example mouse with Calb1^+^ NTS neurons expressing AAV-DIO-hM3Dq-mCherry.

**Supplementary movie S4. Effects of optogenetic activation of Calb1^+^ NTS neurons**, related to Figure 3. Movie S4 shows optogenetic activation of Calb1^+^ NTS neurons with light pulses at 10 Hz and light power 10 mW caused forceful vomiting in an example mouse. The test example mouse with Calb1^+^ NTS neurons expressing AAV-DIO-ChR2-mCherry.

**Supplementary movie S5. GCaMP signal of Calb1^+^ NTS neurons during *B. cereus*-induced retching behavior**, related to Figure 4. Movie S5 shows fiber photometry recording of GCaMP signal of Calb1^+^ NTS neurons during *B. cereus*-induced retching behavior in an example mouse. It can be observed that the peak of GCaMP signal was temporally matched with the retching behavior in mouse.

**Supplementary movie S6. Effects of optogenetic activation of Calb1^NTS-Amb/RVLM^ pathway repeatedly caused forceful vomiting in satiety mice**, related to Figure 5. Movie S6 shows optogenetic activation of Calb1^NTS-Amb/RVLM^ pathway with light pulses at 10 Hz and light power 10 mW caused forceful vomiting in an example satiety mouse (please see method for more information).

**Supplementary movie S7. Effects of optogenetic activation of Calb1^NTS-Amb/RVLM^ pathway caused retching behavior in mice**, related to Figure 5. Movie S7 shows optogenetic activation of Calb1^NTS-Amb/RVLM^ pathway with light pulses at 10 Hz and light power 20 mW evoked intense retching behavior in an example mouse (top). Gastric pressure, EMG and mouth opening angle were recorded during retching (bottom).
