# Supplementary material for: Brain circuits for retching-like behavior
Source: Natl Sci Rev. 2023 Sep 27;11(1):nwad256. doi: 10.1093/nsr/nwad256 (PMC10824557; doi:10.1093/nsr/nwad256)
Supplement: nwad256_Supplemental_Files [file nwad256_supplemental_files.zip › Supplementary Table 1 Information of mouse and reagents-20230904.docx]

| **Supplementary Table 1 Information of mouse lines and reagents** | | |
| --- | --- | --- |
| **Mouse Lines** | | |
| *FosCreER* mice | JAX Mice | Stock No: 021882 |
| *vGlut2*-ires-Cre | JAX Mice | Stock No: 016963 |
| *GAD2*-ires-Cre | JAX Mice | Stock No: 028867 |
| *Tac1*-ires-Cre | JAX Mice | Stock No: 021877 |
| *Calb1*-2A-Cre | JAX Mice | Stock No: 028532 |
| *Etv1*-CreER | JAX Mice | Stock No: 013048 |
| *Dbh*-2A-Flp | UCD | Stock No: 041575 |
| *Chat*-cre | JAX Mice | Stock No: 006410 |
| **Antibodies** | | |
| Rabbit polyclonal anti-EGFP | Abcam | ab290 |
| Chicken polyclonal anti-EGFP | Abcam | ab13970 |
| Chicken polyclonal anti-mCherry | Abcam | ab205402 |
| Rabbit Polyclonal Anti-Glutamate | Sigma | G6642 |
| Rabbit Polyclonal Anti-GABA | Sigma | A2052 |
| Rabbit monoclonal Anti-Fos | Cell Signaling Technology | 2250 |
| Mouse monoclonal anti-CGRP | Abcam | ab81887 |
| Rabbit polyclonal anti-vAChT | Synaptic System | 139103 |
| Rabbit polyclonal anti-Calbindin1 | Swant | CB-38 |
| Rabbit polyclonal anti-NeuN | Abcam | ab177487 |
| Goat polyclonal anti-Choline Acetyltransferase | Merck-Millipore | ab144p |
| Rabbit polyclonal anti-RFP | Abcam | ab62341 |
| Goat Anti-Mouse IgG H&L (FITC) | Abcam | ab6785 |
| Goat anti-Rabbit IgG (H+L) Alexa 488 | Thermo Fisher Scientific | A11034 |
| Donkey anti-Rabbit IgG (H&L) Alexa 488 | Abcam | ab150073 |
| Goat anti-Mouse IgG (H+L) Alexa 488 | Thermo Fisher Scientific | A11001 |
| Goat anti-Rabbit IgG (H+L) Alexa 546 | Thermo Fisher Scientific | A11010 |
| Goat anti-Chicken IgY (H+L) Alexa 546 | Thermo Fisher Scientific | A11040 |
| Goat anti-Mouse IgG (H+L) Alexa 546 | Thermo Fisher Scientific | A11030 |
| Donkey anti-Goat IgG (H&L) Alexa 555 | Abcam | ab150130 |
| Goat anti-Rabbit IgG (H+L) Alexa 633 | Thermo Fisher Scientific | A21071 |
| Goat anti-Mouse IgG (H+L) Alexa 647 | Thermo Fisher Scientific | A21235 |
| **Chemicals, peptides, and recombinant proteins** | | |
| D-AP5 | Tocris | 0106 |
| CNQX | Tocris | 0190 |
| Picrotoxin | Tocris | 1128 |
| TTX | Tocris | 1078 |
| 4-AP | Tocris | 0940 |
| DAPI | Solarbio | C0065 |
| CTB-555 | Thermo Fisher Scientific | C34775 |
| CTB-488 | Thermo Fisher Scientific | C34776 |
| Cereulide | Chiralix | CX20422 |
| 4-Hydroxytamoxifen | Sigma | H6278 |
| Clozapine N-oxide | ENZO | BML-NS105 |
| Dispase® II | Roche | 04942078001 |
| Collagenase type 2 | worthington | LS004176 |
| SEA | Toxin Technology | AT101 |
| **Bacterial and virus strains** | | |
| AAV2/9-hSyn-DIO-hM3Dq-mCherry | TaiToll | N/A |
| AAV2/9-nEF1a-fDIO-hM3D(Gq)-EGFP | BrainVTA | N/A |
| AAV2/9-hEF1a-DIO-hChR2-mCherry | TaiToll | N/A |
| AAV2/9-EF1a-DIO-hChR2-EYFP | BrainVTA | N/A |
| scAAV2/1-hSyn-Cre | TaiToll | N/A |
| AAV2/9-nEF1α-fDIO-hChR2-EYFP | BrainVTA | N/A |
| PRV-CAG-EGFP | BrainVTA | N/A |
| AAV2/9-Ef1a-DIO-mCherry | BrainVTA | N/A |
| AAV2/9-EF1a-fDIO-RV-G | BrainVTA | N/A |
| RV-EnvA-DG-DsRed | BrainVTA | N/A |
| rAAV-EF1α-DIO-H2B-EGFP | BrainVTA | N/A |
| rAAV-EF1α-DIO-H2B-EGFP-mrubby | BrainVTA | N/A |
| AAV2/9-hSyn-DIO-hM4Di-mCherry | TaiToll | N/A |
| AAV2/9-hSyn-hM4D(Gi)-mCherry | TaiToll | N/A |
| AAV2/9-EF1a-DIO-eGFP-Syb2 | TaiToll | N/A |
| AAV2/9-hSyn-DIO-mCherry | TaiToll | N/A |
| AAV2/Retro-EF1a-DIO-mCherry | BrainVTA | N/A |
| AAV2/retro-EF1a-DIO-EGFP | BrainVTA | N/A |
| AAV2/9-CAG-DIO-GCaMp6s | BrainVTA | N/A |
| AAV2/retro-DIO-flp | BrainVTA | N/A |
| AAV2/2Retro Plus-hEF1a-mCherry-Cre | TaiToll | N/A |
| AAV2/9-hSyn-mEGFP | BrainVTA | N/A |
