# Supplementary material for: Brain circuits for retching-like behavior
Source: Natl Sci Rev. 2023 Sep 27;11(1):nwad256. doi: 10.1093/nsr/nwad256 (PMC10824557; doi:10.1093/nsr/nwad256)
Supplement: nwad256_Supplemental_Files [file nwad256_supplemental_files.zip › Supplementary Table 2 Summary of all experimental designs-20230904.docx]

| **Supplementary Table 2 Summary of all experimental designs** | | | | |
| --- | --- | --- | --- | --- |
| **Figures** | **Aims** | **Mouse lines** | **AAV injection & optical fiber implantation** | **Type of data** |
| Fig.1a-1d  Fig. S1a-1d | Quantitative analyses of retching induced by B. cereus | *WT* | No AAV injection. No optical fiber implantation | Behavior |
| Fig.1e | Identify nuclei associated with retching | *WT* | No AAV injection. No optical fiber implantation | Behavior  Histology |
| Fig.1f-1g  Fig.S1e-1g | FosTRAP experiments to label NTS neurons of retching induced by B. cereus  Validating the efficiency and specificity of hM3D to activate NTS neurons | *FosCreER* | AAV-EF1a-DIO-hM3D(Gq)-mCherry injected into the NTS;  AAV-EF1a-DIO-mCherry injected into the NTS; | Behavior  Slice physiology  Histology |
| Fig.1h-1i  Fig.S2d | Cell-type-specific activation of NTS neurons | vGlut2-ires-Cre  *GAD-ires*-Cre  *Chat*-cre | AAV2/9-hEF1a-DIO-hChR2-mCherry injected into the NTS, Optical fiber implanted above the NTS | Behavior  Histology |
| Fig.2a-f  Fig.S2a-c  Fig. S3a-3d | NTS single-nucleus RNA sequencing | *WT* | No AAV injection. No optical fiber implantation | Analysis of RNA sequencing |
| Fig.2g | Distribution of subtype markers in NTS neurons | *Calb1*-Cre  *Tac1* -cre  *Etv1*CreER  *DBH*-Flp | AAV-DIO/fDIO-H2B-EGFP | Histology |
| Fig. 3a-g  Fig.S4a-4f | Activation of NTS neurons subtypes | *Calb1*-Cre  *Tac1* -cre  *Etv1*CreER  *DBH*-flp | AAV-DIO/fDIO-hM3D(Gq)-mCherry or AAV-DIO/fDIO-mCherry injected into the NTS;  AAV2/9-DIO/fDIO-hChR2-mCherry or AAV-DIO/fDIO-mCherry injected into the NTS, Optical fiber implanted above the NTS | Behavior  Histology  Slice physiology |
| Fig.3h  Fig.S5 | Colocalization of *B. cereus* TRAPed NTS neurons with Calb1^+^ neurons or Tac1^+^ neurons; Colocalization of Tac1^+^ and Calb1^+^ neurons in NTS. | *FosCreER*  *WT*  *Tac1-cre* | AAV-DIO-hM3D-mCherry injected into the NTS  AAV-DIO-H2B-EGFP in NTS | Histology |
| Fig.3i-j | Inactivation Calb1 neurons of NTS | *Calb1*-Cre | AAV2/9-hSyn-DIO-hM4Di-mCherry or AAV2/9-hSyn-DIO -mCherry injected into the NTS | Behavior  Histology  Slice physiology |
| Fig4a-c | NTS Calb1 neurons Electrophysiological properties | *Calb1*-Cre | AAV-Ef1α-DIO-EGFP injected into the NTS | Slice physiology |
| Fig.4d-e  Fig.S6 | Recording Calb1^+^ neuronal activity in retching mice | *Calb1*-Cre  Tac1-cre | AAV2/9-CAG-DIO-GCaMp6s or AAV-DIO-EGFP injected into the NTS Optical fiber implanted above the NTS | Fiber photometry |
| Fig.4f-i  Fig.S7a-d  Fig. 5d | Anterograde tracing downstream pathway; Retrograde tracing of NTS-PBNel and NTS-Amb pathway. | *Calb1*-Cre | AAV-hSyn-DIO-H2B-EGFP-mRuby  AAV-DIO-EGFP-syb2 injected into the NTS  AAV2/2Retro-Ef1α-DIO-mCherry  AAV2/2Retro-Ef1α-DIO-EGFP (Injection strategy see Fig. 3B)  CTB555 injected into the Amb or PBNel  PRV injected into diaphragm | Histology |
| Fig.5a-c  Fig. 5e  Fig.4j-h  Fig.S8a-8e | Activation of NTS-Amb and NTS-PBNel pathways | *Calb1*-Cre | AAV2/9-hEF1a-DIO-hChR2-mCherry or AV2/9-hEF1a-DIO -mCherry injected into the NTS, Optical fiber implanted above the PBNel or Amb | Histology  Behavior  Slice physiology |
| Fig.5f-g  Fig. 8f | Inactivation of NTS-Amb pathways | *Calb1*-Cre | AAV2/9-hEF1a-DIO-hChR2-mCherry injected into the NTS, AAV-hsyn- hM4Di-mCherry, Optical fiber implanted above Amb | Behavior  Histology |
| Fig. S9 | Cereulide induced retching behavior | *WT* | No AAV injection. No optical fiber implantation | Behavior  Histology |
| Fig.6a | Effect of Cereulide on excitability of JNG neurons | *WT* | No AAV injection. No optical fiber implantation | Electrophysiological recording |
| Fig.6b-d  Fig.S10 | RV tracing of projecting Calb1+ NTS neurons | *Calb1*-Cre | AAV-DIO-EGFP-T2A-TVA and AAV-DIO-RVG in NTS, RV-EvnA-△G-DsRed in NTS (Injection strategy see Fig. 6b) | Histology |
| Fig.6e-g  Fig.S11  Fig.S12  Fig.S13 | JNG neurons single cell RNA sequence | *Calb1*-Cre  *WT* | RV in NTS (Injection strategy see Fig. 6b)  No AAV injection. No optical fiber implantation | Analysis of RNA sequencing |
