# Supplementary material for: Brain circuits for retching-like behavior
Source: Natl Sci Rev. 2023 Sep 27;11(1):nwad256. doi: 10.1093/nsr/nwad256 (PMC10824557; doi:10.1093/nsr/nwad256)
Supplement: nwad256_Supplemental_Files [file nwad256_supplemental_files.zip › Supplementary Table 3 Summary of Cell Counting Strategy.docx]

| **Supplementary Table 3 Summary of Cell Counting Strategy** | | | |
| --- | --- | --- | --- |
| Brain region | Nodose | NTS | The whole brain |
| Section type | Coronal section (15 μm) | Coronal section (40 μm) | Coronal section (40 μm) |
| Section Range | The whole nodose | Bregma (-7.32 to -7.52) | The whole brain |
| Total collection | Approximately 30 sections | Approximately 5 sections | Approximately 300 sections |
| Sampling | To sample 1 section every 3 sections to get 6 sections evenly spaced by the whole nodose | To sample 1 section every 5 sections to get 4 sections evenly spaced by 200 μm | To sample 1 section every 5 sections to get 50 sections evenly spaced by the whole brain |
