# Supplementary material for: Brain circuits for retching-like behavior
Source: Natl Sci Rev. 2023 Sep 27;11(1):nwad256. doi: 10.1093/nsr/nwad256 (PMC10824557; doi:10.1093/nsr/nwad256)
Supplement: nwad256_Supplemental_Files [file nwad256_supplemental_files.zip › Supplementary Table 4 Summary of statistical analyses-0905.docx]

| **Supplementary Table 4. Summary of statistical analyses** | | | |
| --- | --- | --- | --- |
| Figure | Sample size（n） | Statistical test | P values |
| 1c | Vehicle:  n = 11 mice  *B. Cereus*:  n = 10 mice | Student t-test | Bacillus vs. Vehicle :P < 0.0001 **** |
| 1g | Saline: Ctrl n=4 mice, hM3D n=4 mice; CNO: n=4 mice  n = 5 mice | Student t-test | CNO Group: Ctrl vs. hM3D P=0.0003 *** |
| 1i | GAD-Cre: n = 4 mice  vGlut2-Cre: n = 4 mice | Two-way ANOVA | 10mW：  GAD-Cre vs vGlut2-Cre: P=0.0011 **  20mW：  GAD-Cre vs vGlut2-Cre: P<0.0001 **** |
| 3a | n = 4 mice/line | One-way ANOVA | \| *Etv1*-CreER vs. *DBH*-2A-Flp: P > 0.9999 n.s \| \| --- \| \| *Etv1*-CreER vs. *Tac1*-ires-Cre: P = 0.0087 ** \| \| *Etv1*-CreER vs. *Calb1*-2A-Cre: P = 0.0023 ** \| \| *DBH*-2A-Flp vs. *Tac1*-ires-Cre: P = 0.00876 ** \| \| *DBH*-2A-Flp vs. *Calb1*-2A-Cre: P = 0.0023 ** \| \| *Tac1*-ires-Cre vs. *Calb1*-2A-Cre: P = 0.9741 n.s. \| |
| 3d | n = 4 mice/line | Two-way ANOVA | Optogenetic activation 5mW, *Tac1*-ires-Cre vs. *Calb1*-2A-Cre: P = 0.0585 n.s  Optogenetic activation 10mW, *Tac1*-ires-Cre vs. *Calb1*-2A-Cre: P = 0.0174 *  Optogenetic activation 20mW, *Tac1*-ires-Cre vs. *Calb1*-2A-Cre: P = 0.0174 * |
| 3e | n = 16 mice | Student t-test | Retching numbers / 5s:   \| 5Hz vs. 10 Hz: P < 0.0001 **** \| \| --- \| \| 5 Hz vs. 20 Hz: P < 0.0001**** \| \| 10 Hz vs. 20 Hz: P < 0.0001**** \| |
| 3i | Saline: Ctrl n=5 mice, hM4D n = 4 mice;  CNO: Ctrl n=6 mice, hM4D n = 7 mice | Student t-test | Retching numbers / 1h:  Saline: Ctrl vs. hM4D: P=0.7051 n.s  CNO: Ctrl vs. hM4D: P=0.0006 *** |
| 4b | n = 7cells | Paired t-test | Amplitude of PSCs:   \| Before vs. PTX: P = 0.8137 n.s \| \| --- \| \| Before vs. APV+CNQX: P = 0.0002 *** \| |
| 5a Right | n = 5 cells | Paired t-test | Amplitude of PSCs:   \| Before vs. +BIC: P = 0.9968 n.s \| \| --- \| \| Before vs. +APV and CNQX: P = 0.0024 ** \| |
| 5a Left | n = 9 cells | Paired t-test | \| Before vs. +BIC: P = 0.9962 \| \| --- \| \| Before vs. +APV and CNQX: P < 0.0001 **** \| |
| 5b | LPB: n = 18mice  Amb: n = 18mice | Two-way ANOVA | Laser intensity Retching numbers   \| Amb: \| \| --- \| \| 10mW vs. 5 mW: P < 0.0001 **** \| \| 10 mW vs. 20 mW: P = 0.0022  Lase frequency Retching numbers   \| Amb: \| \| --- \| \| 10Hz vs. 5 Hz: P < 0.0001 **** \| \| 10 Hz vs. 20 Hz: P < 0.0001 **** \| \| |
| 5c | mCherry: n = 6 mice  ChR2: n = 6 mice | Student t-test | Weight of vomitus ChR2 vs. mCherry: P = 0.0132 * |
| 5g | Saline: n = 8 mice  CNO: n = 8 mice | Two-way ANOVA | Retching numbers / 5s:  5mW: Saline vs. CNO: P < 0.0001 ****  20mW: Saline vs. CNO: P < 0.0001 ****  Saline 5mW vs. Saline 20mW: P = 0.0028 ** |
| 5i | Control: n = 8 mice  NTS-Amb: n = 10 mice  NTS-PBN: n = 9 mice | Two-way ANOVA | Preference index:   \| Baseline: Control vs. NTS-Amb: P = 0.9976 n.s \| \| --- \| \| Control vs. NTS-PBN: P = 0.8241 n.s  NTS-Amb vs. NTS-PBN: P = 0.8417 n.s \| \| Condition: Control vs. NTS-Amb: P = 0.0002 *** \| \| Control vs. NTS-PBN: P = 0.0005 ***  NTS-Amb vs. NTS-PBN: P = 0.9878 n.s \| \| Test: Control vs. NTS-Amb: P=0.0259 * \| \| Control vs. NTS-PBN: P=0.0136*  NTS-Amb vs. NTS-PBN: P = 0.9450 n.s \| |
| 6a top | Type III-activated:  n = 10 cells  Type II-inhibited:  n = 12 cells  Type I-no response:  n = 8 cells | Student t-test | Type III-activated: + Cereulide vs. Before: P = 0.0259 *  Type II-inhibited: + Cereulide vs. Before: P = 0.0136 *  Type I-no response: + Cereulide vs. Before: P = 0.4512 n.s |
| 6a down | Type I-no response:  n = 9 cells  Type II-inhibited:  n = 12 cells  Type III-activated:  n = 9 cells | Two-way ANOVA | Type I-no response: + Cereulide vs. Before: n.s.  Type II-inhibited: + Cereulide vs. Before:  80 pA P = 0.0327 *  100 Pa P = 0.0069 **  120 pA P = 0.0009 ***  140 pA P = 0.0002 ***  160 pA P < 0.0001 ****  180 pA P < 0.0001 ****  200 pA P < 0.0001 ****  Type III-activated: + Cereulide vs. Before:  80 pA P = 0.035 *  100 pA P = 0.0148 *  120 pA P = 0.0198 *  140 pA P = 0.0264 * |
| S1a | Male: n = 5 mice  Fmale: n = 5 mice | Student t-test | Retching number/3h: Male vs. Fmale: P= 0.6787 n.s |
| S1b | Vehicle: n=18 Trials  *B. Cereus*: n=18 Trials | Student t-test | Vehicle vs. B. Cereus: P= 0.0001 **** |
| S1c | Vehicle: n=18 Trials  *B. Cereus*: n=18 Trials | Student t-test | Vehicle vs. B. Cereus: P= 0.0001 **** |
| S1f | Vehicle: n=6 mice  *B. Cereus*: n=6 mice | Student t-test | Retching number/1h: Bacillus vs. Vehicle: P=0.0009 *** |
| S2d | GAD-cre: n = 4mice  Chat-cre: n = 4mice  vGlut2-cre: n = 4mice | Two-way ANOVA | Retching number/5s:  10mW:   \| GAD-cre vs. vGlut2-cre: P < 0.0001 **** \| \| --- \| \| Chat-cre vs. vGlut2-cre: P < 0.0001 **** \|   20mW:   \| GAD-cre vs. vGlut2-cre: P < 0.0001 **** \| \| --- \| \| Chat-cre vs. vGlut2-cre: P < 0.0001 **** \| |
| S8c | Laser OFF: Ctrl n=10 Trials  ChR2 n=10 Trials;  Laser ON: Ctrl n=10 Trials  ChR2 n=10 Trials | Two-way ANOVA | Laser OFF: Ctrl vs. ChR2: P= 0.9999  Laser ON: Ctrl vs. ChR2: P= 0.0001 **** |
| S8d | Laser OFF: Ctrl n=10 Trials  ChR2 n=10 Trials;  Laser ON: Ctrl n=10 Trials  ChR2 n=10 Trials | Two-way ANOVA | Laser OFF: Ctrl vs. ChR2: P= 0.4574  Laser ON: Ctrl vs. ChR2: P= 0.0001 **** |
| S8e | Laser OFF: Ctrl n=10 Trials  ChR2 n=10 Trials;  Laser ON: Ctrl n=10 Trials  ChR2 n=10 Trials | Two-way ANOVA | Laser OFF: Ctrl vs. ChR2: P= 0.8150  Laser ON: Ctrl vs. ChR2: P= 0.0001 **** |
| S8f | Saline: Ctrl n=8 mice,  hM4D n=8 mice;  CNO: Ctrl n=8 mice,  hM4D n=8 mice | Two-way ANOVA | Saline: Ctrl vs. hM4D: P= 0.3125  CNO: Ctrl vs. hM4D: P= 0.0001 **** |
| S9a | n=5 mice | one-way ANOVA | 50 vs.200: P=0.5126  200 vs.500: P<0.0001**** |
