# Supplementary material for: Brain circuits for retching-like behavior
Source: Natl Sci Rev. 2023 Sep 27;11(1):nwad256. doi: 10.1093/nsr/nwad256 (PMC10824557; doi:10.1093/nsr/nwad256)
Supplement: nwad256_Supplemental_Files [file nwad256_supplemental_files.zip › Supplementary Figure S1-S14/Combine-Supplementary Figure S1-S14-Compressed-0925.pdf]

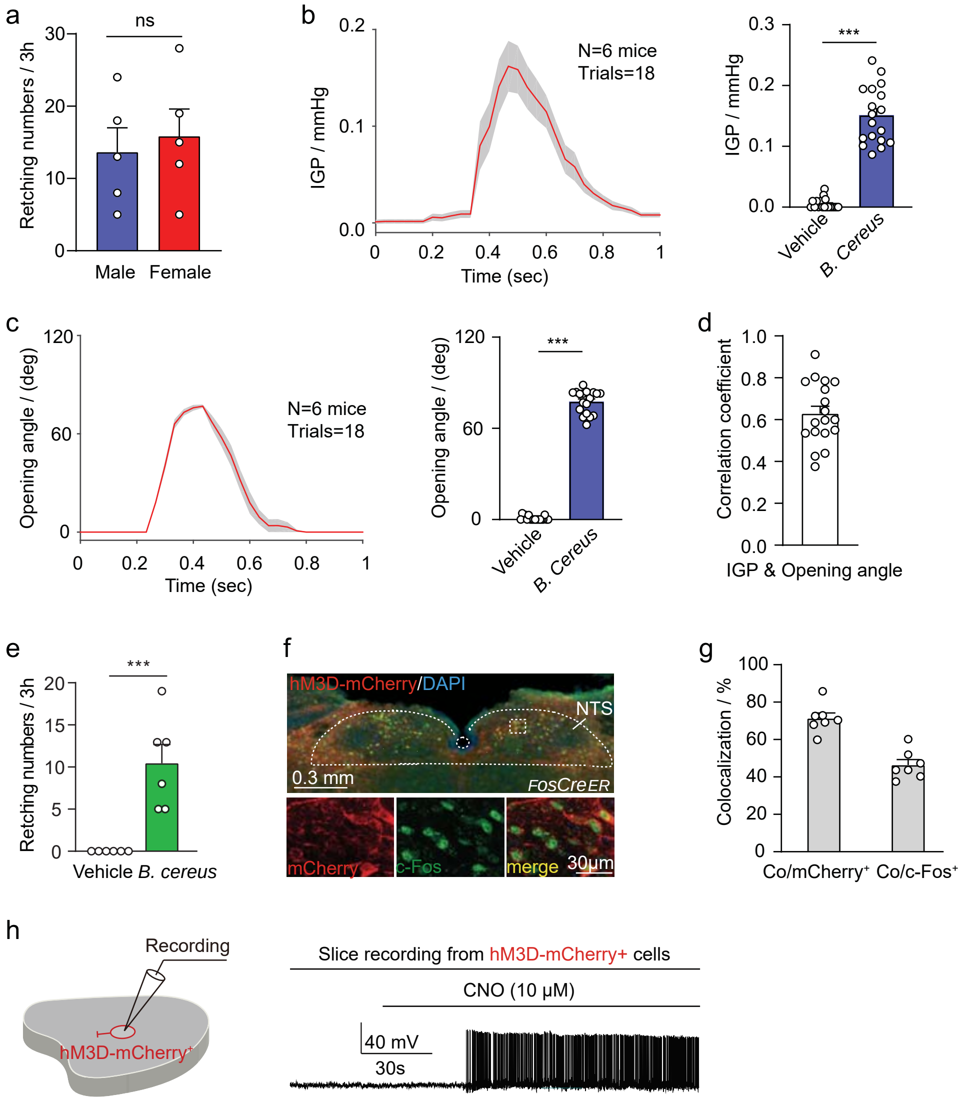
a
ns
Retching numbers / 3h
Male
Female
b
IGP / mmHg
N=6 mice
Trials=18
Time (sec)
***
Vehicle
B. Cereus
c
Opening angle / (deg)
N=6 mice
Trials=18
Time (sec)
***
Vehicle
B. Cereus
d
Correlation coefficient
IGP & Opening angle
e
***
Retching numbers / 3h
Vehicle B. cereus
f
hM3D-mCherry/DAPI
NTS
0.3 mm
FosCreER
mCherry
c-Fos
merge
30μm
g
Colocalization / %
Co/mCherry⁺ Co/c-Fos⁺
h
Recording
hM3D-mCherry⁺
Slice recording from hM3D-mCherry+ cells
CNO (10 μM)
40 mV
30s

Figure S1

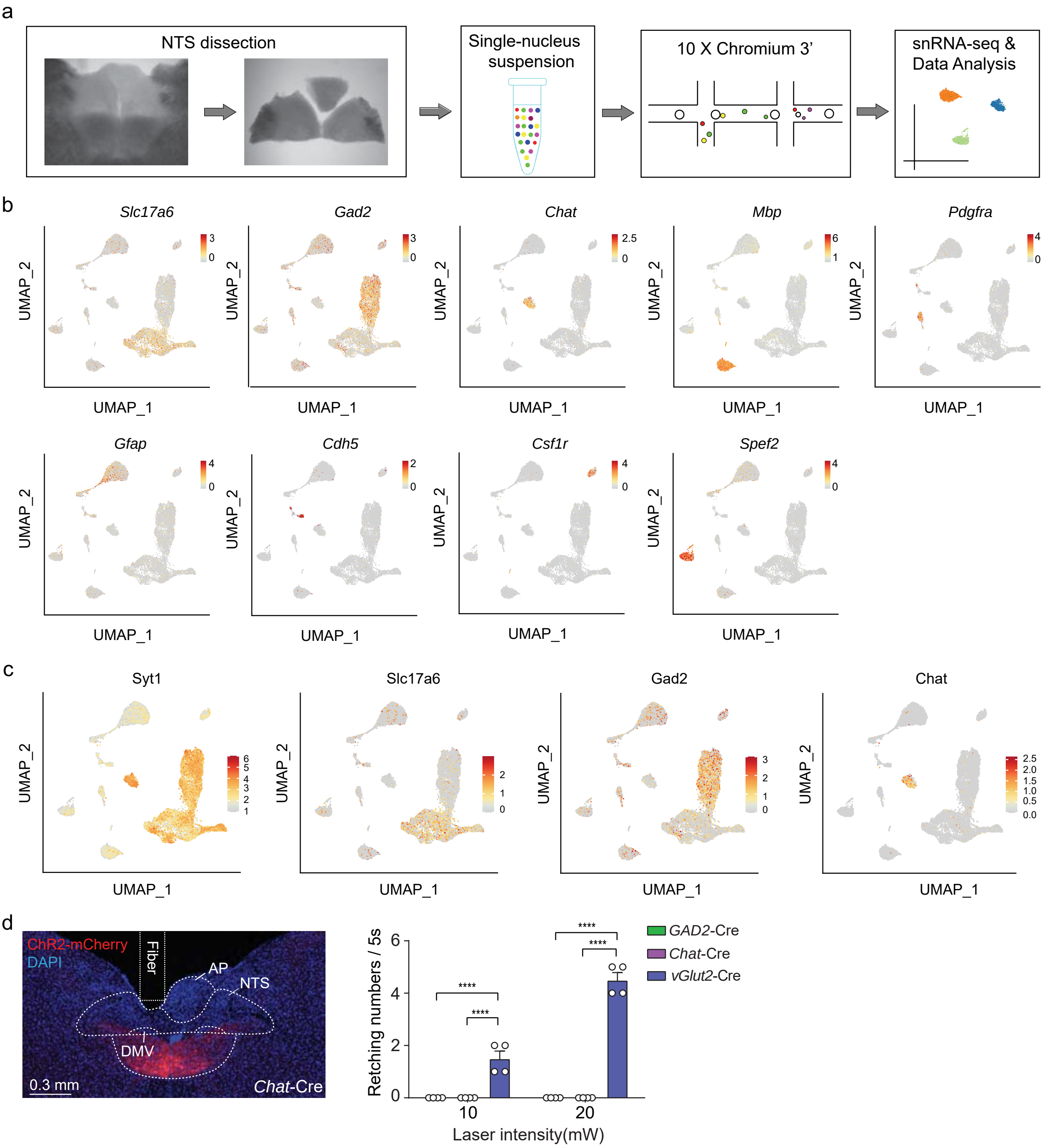
a
NTS dissection
Single-nucleus suspension
10 X Chromium 3’
snRNA-seq & Data Analysis
b
Slc17a6
Gad2
Chat
Mbp
Pdgfra
Gfap
Cdh5
Csf1r
Spef2
UMAP_1
UMAP_2
c
Syt1
Slc17a6
Gad2
Chat
d
ChR2-mCherry
DAPI
Fiber
AP
NTS
DMV
0.3 mm
Chat-Cre
Retching numbers / 5s
Laser intensity(mW)
GAD2-Cre
Chat-Cre
vGlut2-Cre

Figure S2

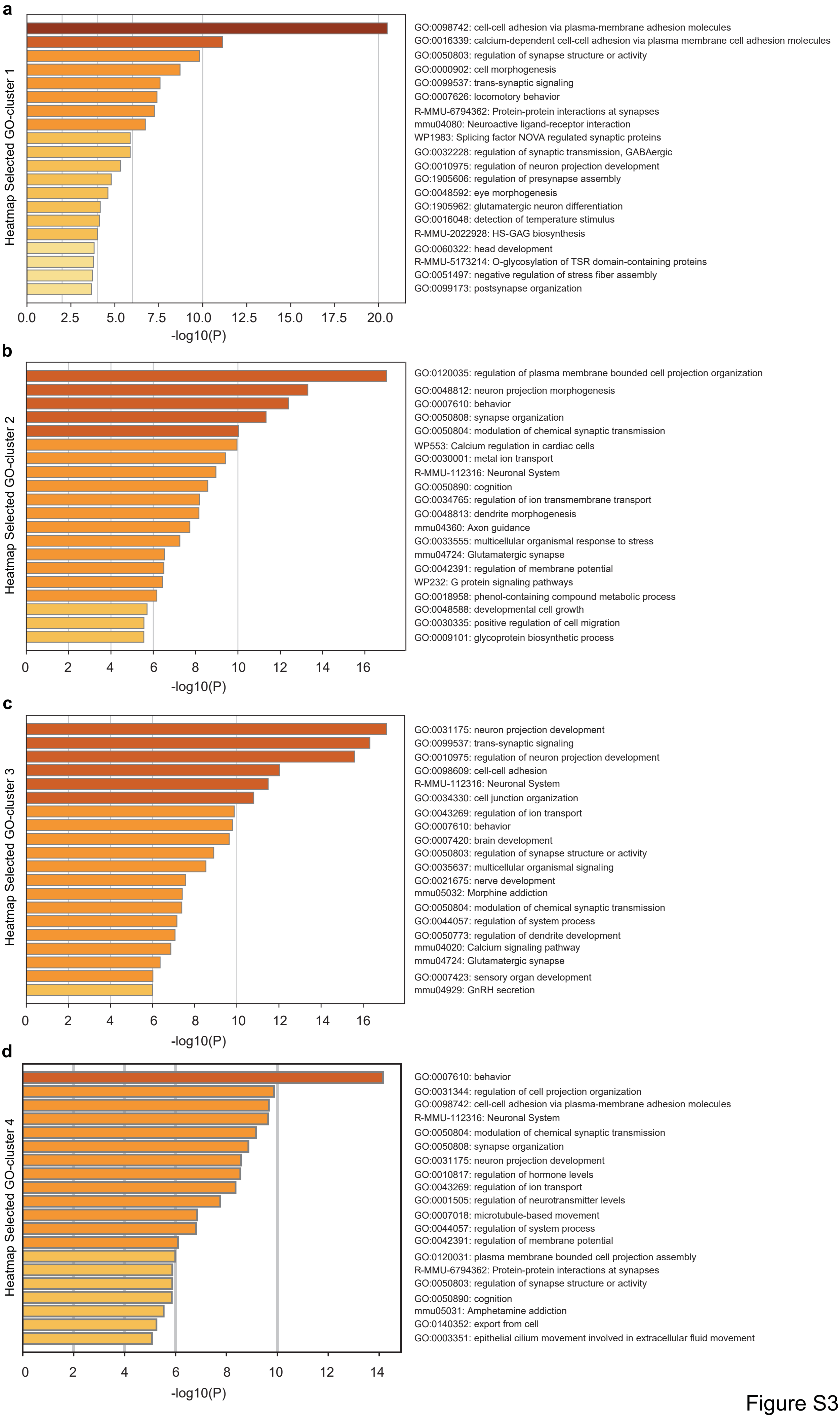
a
Heatmap Selected GO-cluster 1
GO:0098742: cell-cell adhesion via plasma-membrane adhesion molecules
GO:0016339: calcium-dependent cell-cell adhesion via plasma membrane cell adhesion molecules
GO:0050803: regulation of synapse structure or activity
GO:0000902: cell morphogenesis
GO:0099537: trans-synaptic signaling
GO:0007626: locomotory behavior
R-MMU-6794362: Protein-protein interactions at synapses
mmu04080: Neuroactive ligand-receptor interaction
WP1983: Splicing factor NOVA regulated synaptic proteins
GO:0032228: regulation of synaptic transmission, GABAergic
GO:0010975: regulation of neuron projection development
GO:1905606: regulation of presynapse assembly
GO:0048592: eye morphogenesis
GO:1905962: glutamatergic neuron differentiation
GO:0016048: detection of temperature stimulus
R-MMU-2022928: HS-GAG biosynthesis
GO:0060322: head development
R-MMU-5173214: O-glycosylation of TSR domain-containing proteins
GO:0051497: negative regulation of stress fiber assembly
GO:0099173: postsynapse organization
0.0 2.5 5.0 7.5 10.0 12.5 15.0 17.5 20.0
-log10(P)
b
Heatmap Selected GO-cluster 2
GO:0120035: regulation of plasma membrane bounded cell projection organization
GO:0048812: neuron projection morphogenesis
GO:0007610: behavior
GO:0050808: synapse organization
GO:0050804: modulation of chemical synaptic transmission
WP553: Calcium regulation in cardiac cells
GO:0030001: metal ion transport
R-MMU-112316: Neuronal System
GO:0050890: cognition
GO:0034765: regulation of ion transmembrane transport
GO:0048813: dendrite morphogenesis
mmu04360: Axon guidance
GO:0033555: multicellular organismal response to stress
mmu04724: Glutamatergic synapse
GO:0042391: regulation of membrane potential
WP232: G protein signaling pathways
GO:0018958: phenol-containing compound metabolic process
GO:0048588: developmental cell growth
GO:0030335: positive regulation of cell migration
GO:0009101: glycoprotein biosynthetic process
0 2 4 6 8 10 12 14 16
-log10(P)
c
Heatmap Selected GO-cluster 3
GO:0031175: neuron projection development
GO:0099537: trans-synaptic signaling
GO:0010975: regulation of neuron projection development
GO:0098609: cell-cell adhesion
R-MMU-112316: Neuronal System
GO:0034330: cell junction organization
GO:0043269: regulation of ion transport
GO:0007610: behavior
GO:0007420: brain development
GO:0050803: regulation of synapse structure or activity
GO:0035637: multicellular organismal signaling
GO:0021675: nerve development
mmu05032: Morphine addiction
GO:0050804: modulation of chemical synaptic transmission
GO:0044057: regulation of system process
GO:0050773: regulation of dendrite development
mmu04020: Calcium signaling pathway
mmu04724: Glutamatergic synapse
GO:0007423: sensory organ development
mmu04929: GnRH secretion
0 2 4 6 8 10 12 14 16
-log10(P)
d
Heatmap Selected GO-cluster 4
GO:0007610: behavior
GO:0031344: regulation of cell projection organization
GO:0098742: cell-cell adhesion via plasma-membrane adhesion molecules
R-MMU-112316: Neuronal System
GO:0050804: modulation of chemical synaptic transmission
GO:0050808: synapse organization
GO:0031175: neuron projection development
GO:0010817: regulation of hormone levels
GO:0043269: regulation of ion transport
GO:0001505: regulation of neurotransmitter levels
GO:0007018: microtubule-based movement
GO:0044057: regulation of system process
GO:0042391: regulation of membrane potential
GO:0120031: plasma membrane bounded cell projection assembly
R-MMU-6794362: Protein-protein interactions at synapses
GO:0050803: regulation of synapse structure or activity
GO:0050890: cognition
mmu05031: Amphetamine addiction
GO:0140352: export from cell
GO:0003351: epithelial cilium movement involved in extracellular fluid movement
0 2 4 6 8 10 12 14
-log10(P)

Figure S3

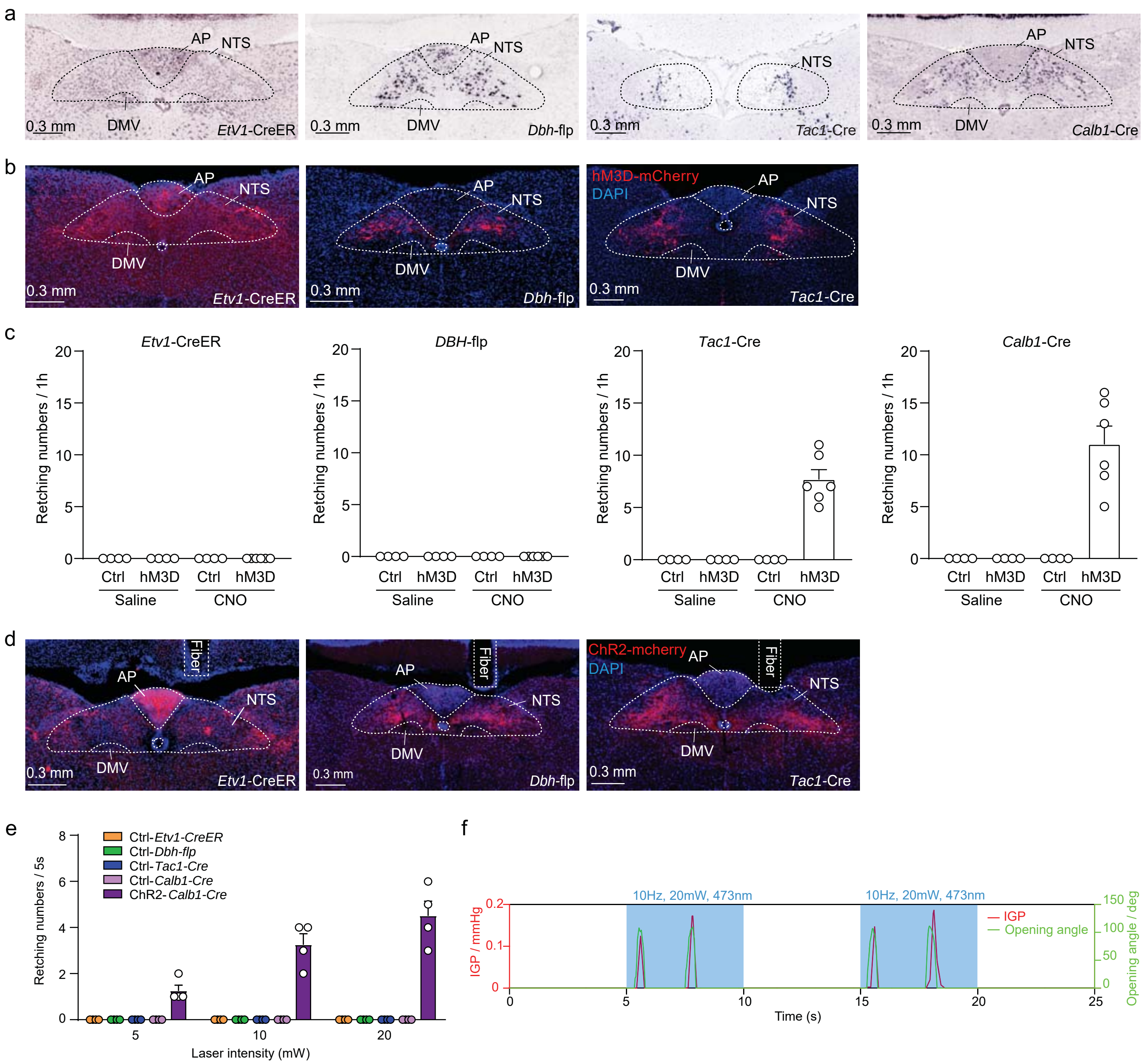
a
AP
NTS
DMV
0.3 mm
EtV1-CreER
Dbh-flp
Tac1-Cre
Calb1-Cre
b
hM3D-mCherry
DAPI
Etv1-CreER
c
Retching numbers / 1h
Etv1-CreER
DBH-flp
Tac1-Cre
Calb1-Cre
Ctrl
hM3D
Saline
CNO
d
Fiber
ChR2-mcherry
e
Ctrl-Etv1-CreER
Ctrl-Dbh-flp
Ctrl-Tac1-Cre
Ctrl-Calb1-Cre
ChR2-Calb1-Cre
Retching numbers / 5s
Laser intensity (mW)
f
10Hz, 20mW, 473nm
IGP / mmHg
IGP
Opening angle
Opening angle / deg
Time (s)

Figure S4

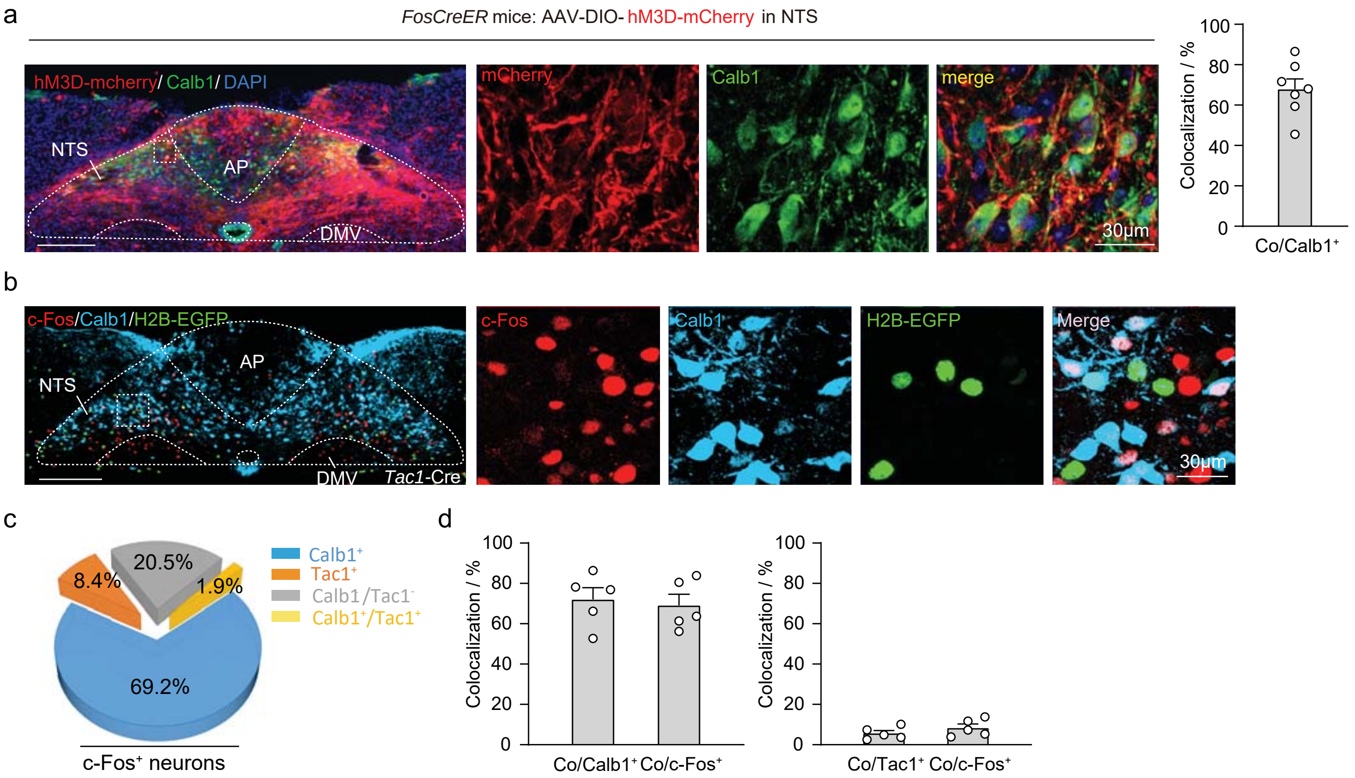
a
FosCreER mice: AAV-DIO- hM3D-mCherry in NTS
hM3D-mcherry/ Calb1/ DAPI
NTS
AP
DMV
mCherry
Calb1
merge
30μm
Colocalization / %
Co/Calb1+
b
c-Fos/Calb1/H2B-EGFP
NTS
AP
DMV
Tac1-Cre
c-Fos
Calb1
H2B-EGFP
Merge
30μm
c
20.5%
8.4%
1.9%
69.2%
Calb1+
Tac1+
Calb1-/Tac1-
Calb1+/Tac1+
c-Fos+ neurons
d
Colocalization / %
Co/Calb1+ Co/c-Fos+
Co/Tac1+ Co/c-Fos+

Figure S5

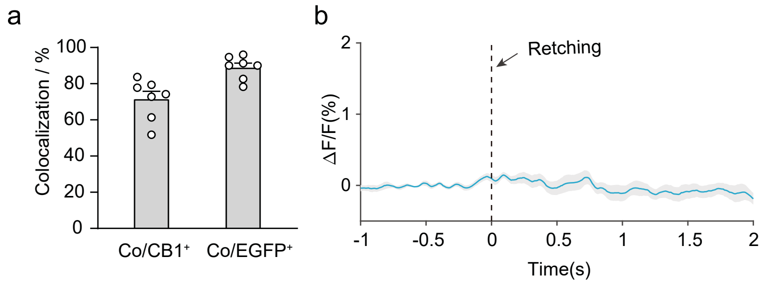
a
Colocalization / %
100
80
60
40
20
0
Co/CB1⁺
Co/EGFP⁺
b
2
1
0
ΔF/F(%)
Retching
-1
-0.5
0
0.5
1
1.5
2
Time(s)

Figure S6

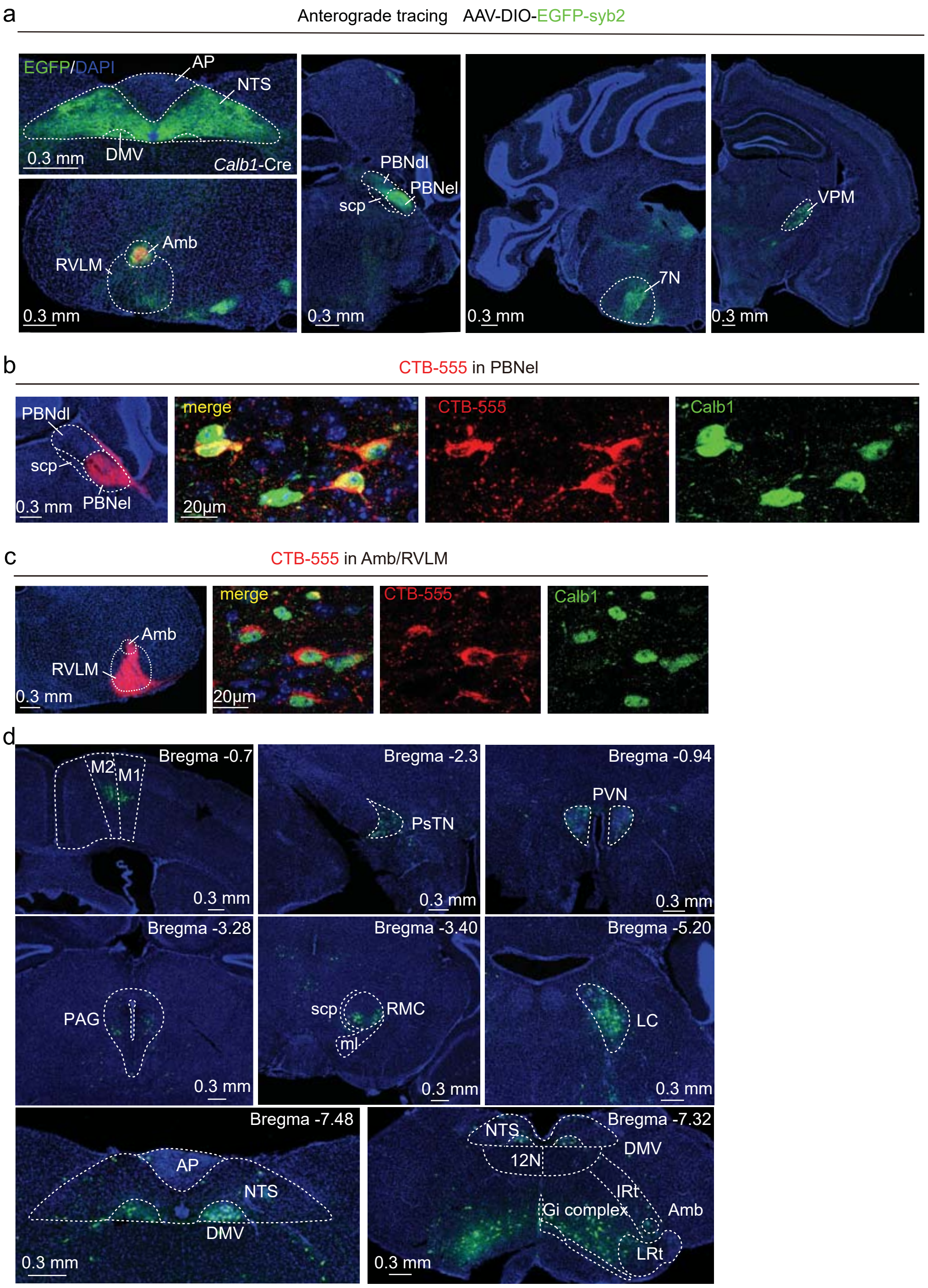
a
Anterograde tracing AAV-DIO-EGFP-syb2
EGFP/DAPI
AP
NTS
DMV
0.3 mm
Calb1-Cre
PBNdl
PBNel
scp
VPM
Amb
RVLM
7N
b
CTB-555 in PBNel
PBNdl
scp
PBNel
merge
CTB-555
Calb1
20μm
c
CTB-555 in Amb/RVLM
Amb
RVLM
d
Bregma -0.7
M2
M1
Bregma -2.3
PsTN
Bregma -0.94
PVN
Bregma -3.28
PAG
Bregma -3.40
scp
RMC
ml
Bregma -5.20
LC
Bregma -7.48
Bregma -7.32
12N
IRt
Gi complex
LRt

Figure S7

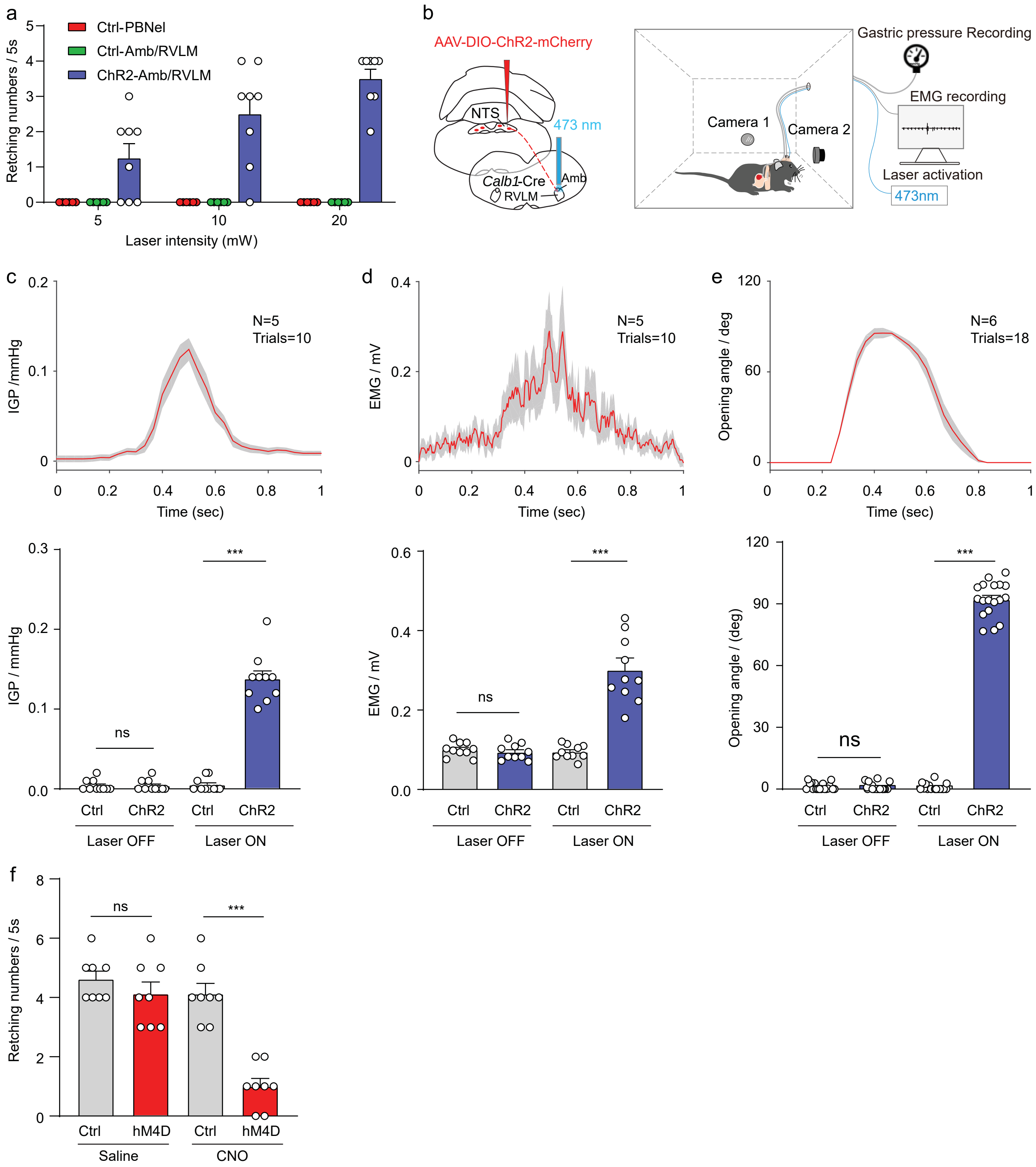
a
Ctrl-PBNel
Ctrl-Amb/RVLM
ChR2-Amb/RVLM
Retching numbers / 5s
Laser intensity (mW)
b
AAV-DIO-ChR2-mCherry
NTS
473 nm
Calb1-Cre
Amb
RVLM
Gastric pressure Recording
EMG recording
Camera 1
Camera 2
Laser activation
473nm
c
N=5
Trials=10
IGP /mmHg
Time (sec)
d
N=5
Trials=10
EMG / mV
Time (sec)
e
N=6
Trials=18
Opening angle / deg
Time (sec)
IGP / mmHg
ns
***
Ctrl
ChR2
Laser OFF
Laser ON
EMG / mV
Opening angle / (deg)
f
Retching numbers / 5s
Ctrl
hM4D
Saline
CNO

Figure S8

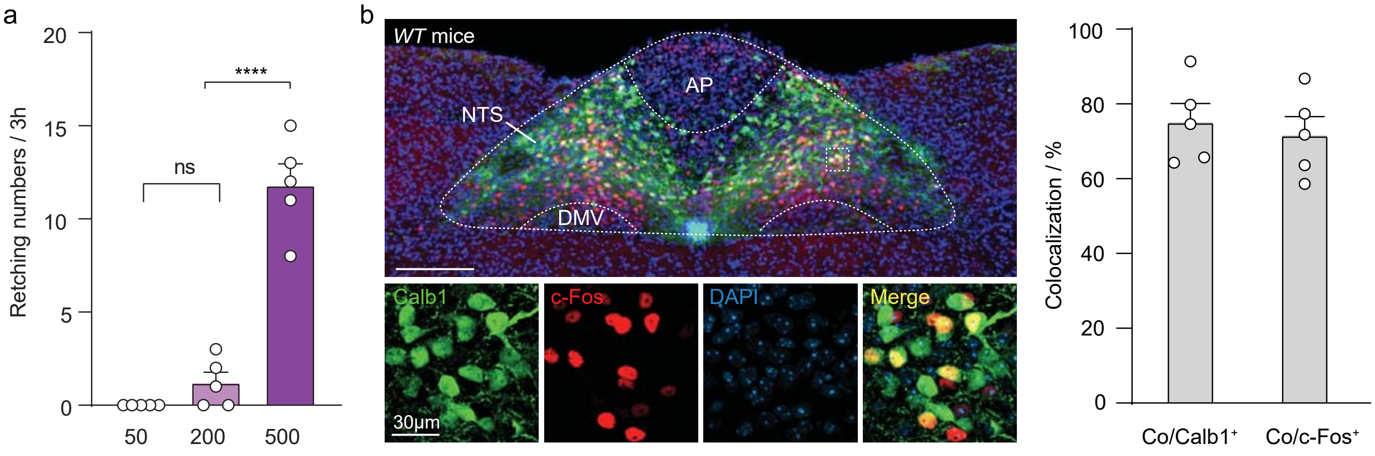
a
Retching numbers / 3h
20
15
10
5
0
50
200
500
ns
****
b
WT mice
AP
NTS
DMV
Calb1
c-Fos
DAPI
Merge
30μm
Colocalization / %
100
80
60
40
20
0
Co/Calb1⁺
Co/c-Fos⁺

Figure S9

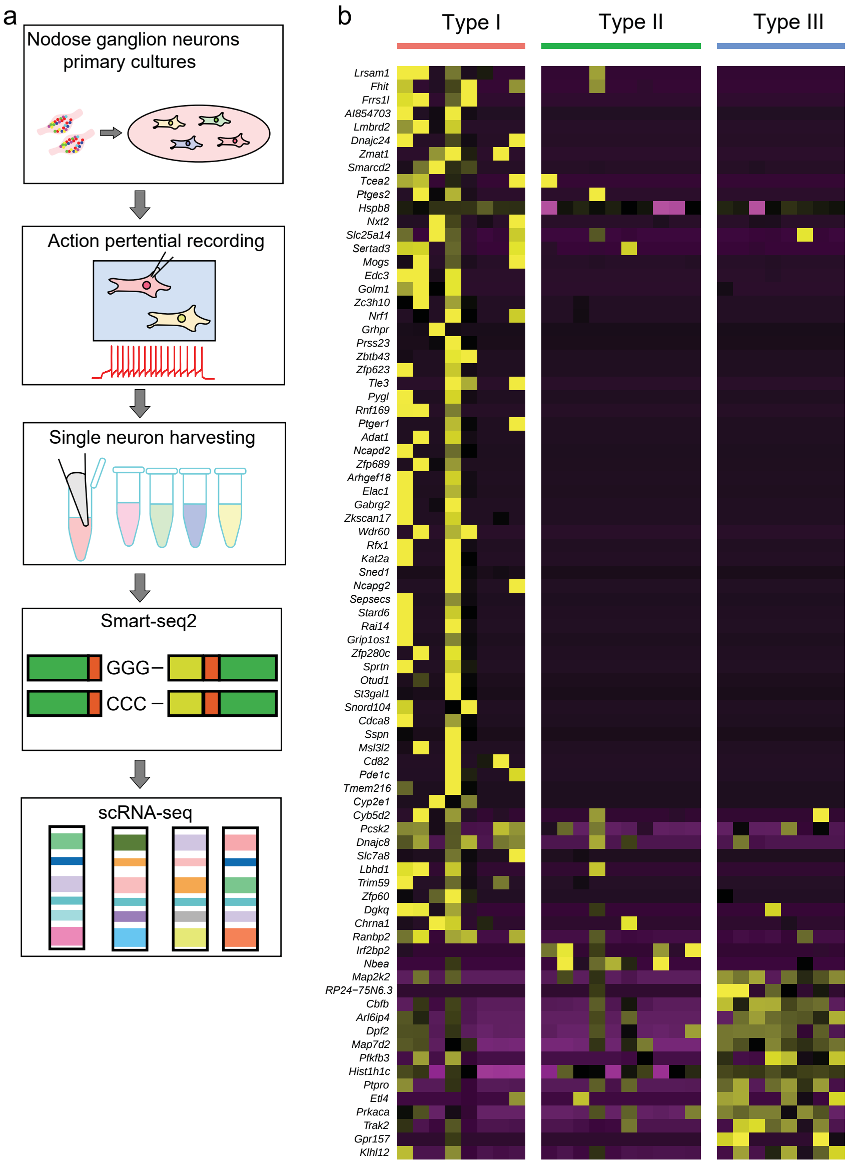
a
Nodose ganglion neurons primary cultures
Action pertential recording
Single neuron harvesting
Smart-seq2
GGG−
CCC −
scRNA-seq
b
Type I
Type II
Type III
Lrsam1
Fhit
Frrs1l
AI854703
Lmbrd2
Dnajc24
Zmat1
Smarcd2
Tcea2
Ptges2
Hspb8
Nxt2
Slc25a14
Sertad3
Mogs
Edc3
Golm1
Zc3h10
Nrf1
Grhpr
Prss23
Zbtb43
Zfp623
Tle3
Pygl
Rnf169
Ptger1
Adat1
Ncapd2
Zfp689
Arhgef18
Elac1
Gabrg2
Zkscan17
Wdr60
Rfx1
Kat2a
Sned1
Ncapg2
Sepsecs
Stard6
Rai14
Grip1os1
Zfp280c
Sprtn
Otud1
St3gal1
Snord104
Cdca8
Sspn
Msl3l2
Cd82
Pde1c
Tmem216
Cyp2e1
Cyb5d2
Pcsk2
Dnajc8
Slc7a8
Lbhd1
Trim59
Zfp60
Dgkq
Chrna1
Ranbp2
Irf2bp2
Nbea
Map2k2
RP24−75N6.3
Cbfb
Arl6ip4
Dpf2
Map7d2
Pfkfb3
Hist1h1c
Ptpro
Etl4
Prkaca
Trak2
Gpr157
Klhl12

Figure S10

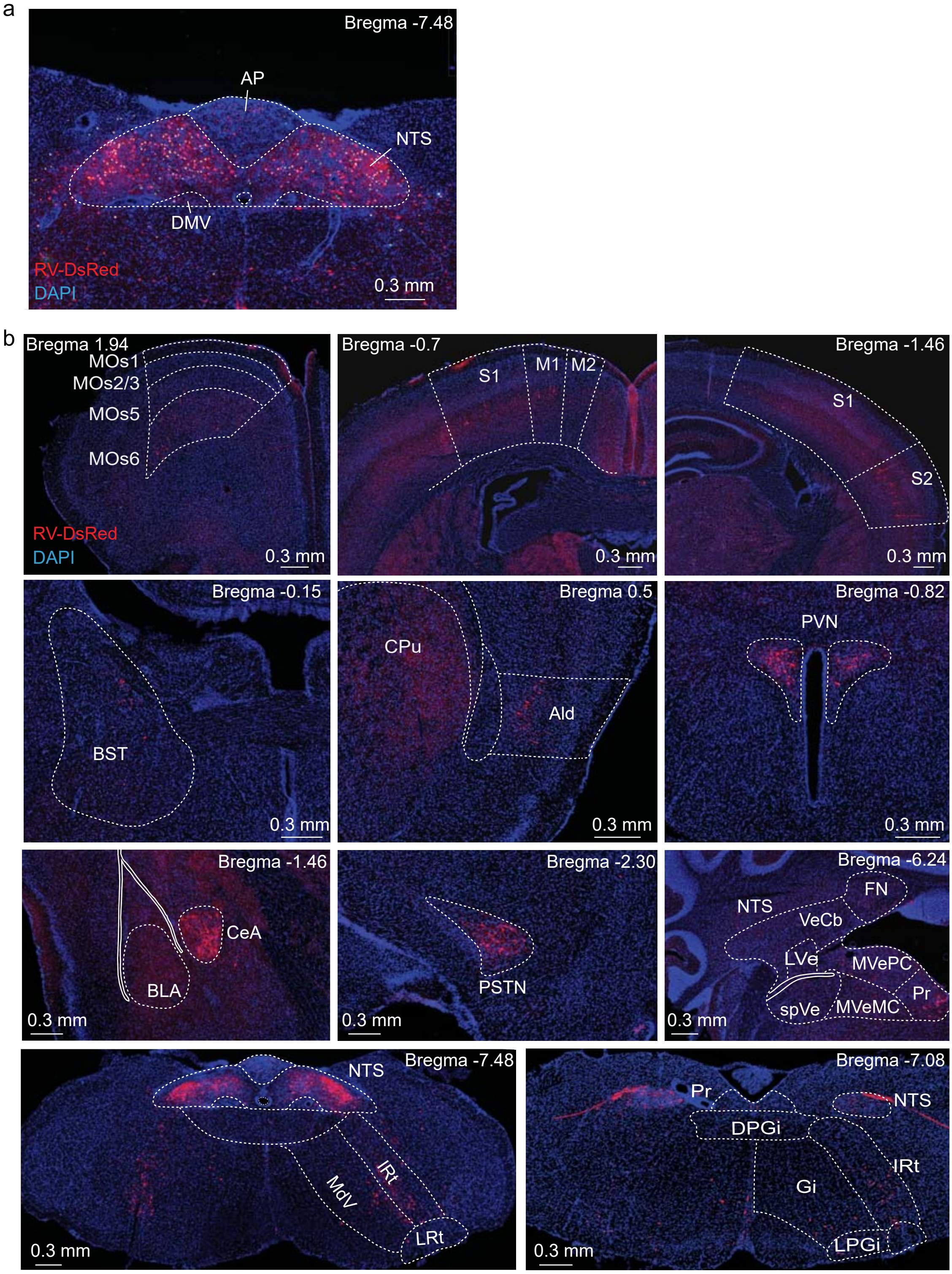
a
Bregma -7.48
AP
NTS
DMV
RV-DsRed
DAPI
0.3 mm
b
Bregma 1.94
MOs1
MOs2/3
MOs5
MOs6
RV-DsRed
DAPI
0.3 mm
Bregma -0.7
S1
M1
M2
0.3 mm
Bregma -1.46
S1
S2
0.3 mm
Bregma -0.15
BST
0.3 mm
Bregma 0.5
CPu
AId
0.3 mm
Bregma -0.82
PVN
0.3 mm
Bregma -1.46
CeA
BLA
0.3 mm
Bregma -2.30
PSTN
0.3 mm
Bregma -6.24
NTS
FN
VeCb
LVe
MVePC
Pr
spVe
MVeMC
0.3 mm
Bregma -7.48
NTS
IRt
MdV
LRt
0.3 mm
Bregma -7.08
Pr
NTS
DPGi
IRt
Gi
LPGi
0.3 mm

Figure S11

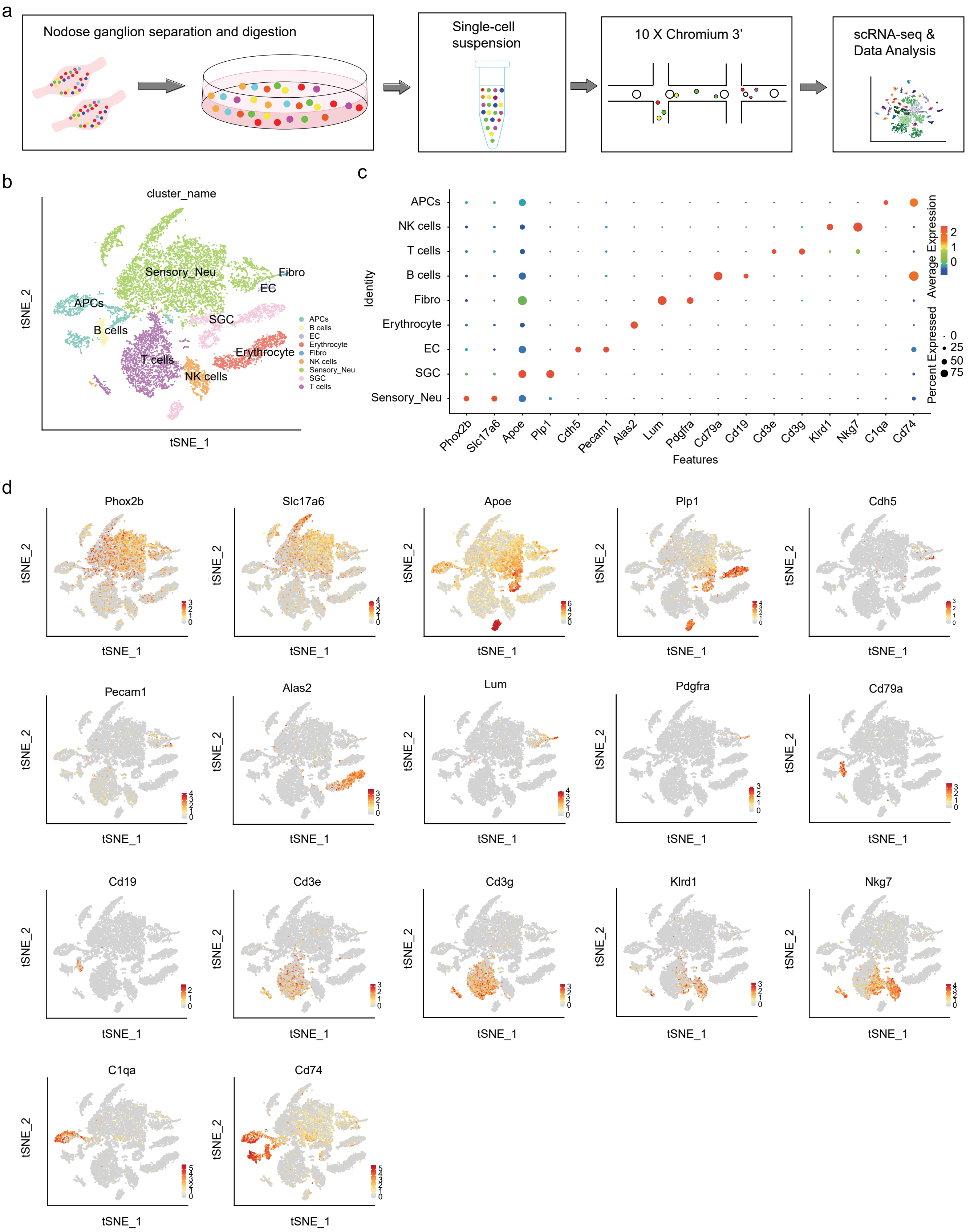
a
Nodose ganglion separation and digestion
Single-cell suspension
10 X Chromium 3'
scRNA-seq & Data Analysis
b
cluster_name
Sensory_Neu
Fibro
EC
APCs
SGC
B cells
T cells
Erythrocyte
NK cells
tSNE_1
tSNE_2
c
APCs
NK cells
T cells
B cells
Fibro
Erythrocyte
EC
SGC
Sensory_Neu
Identity
Phox2b
Slc17a6
Apoe
Plp1
Cdh5
Pecam1
Alas2
Lum
Pdgfra
Cd79a
Cd19
Cd3e
Cd3g
Klrd1
Nkg7
C1qa
Cd74
Features
Average Expression
Percent Expressed
d
Phox2b
Slc17a6
Apoe
Plp1
Cdh5
Pecam1
Alas2
Lum
Pdgfra
Cd79a
Cd19
Cd3e
Cd3g
Klrd1
Nkg7
C1qa
Cd74

Figure S12

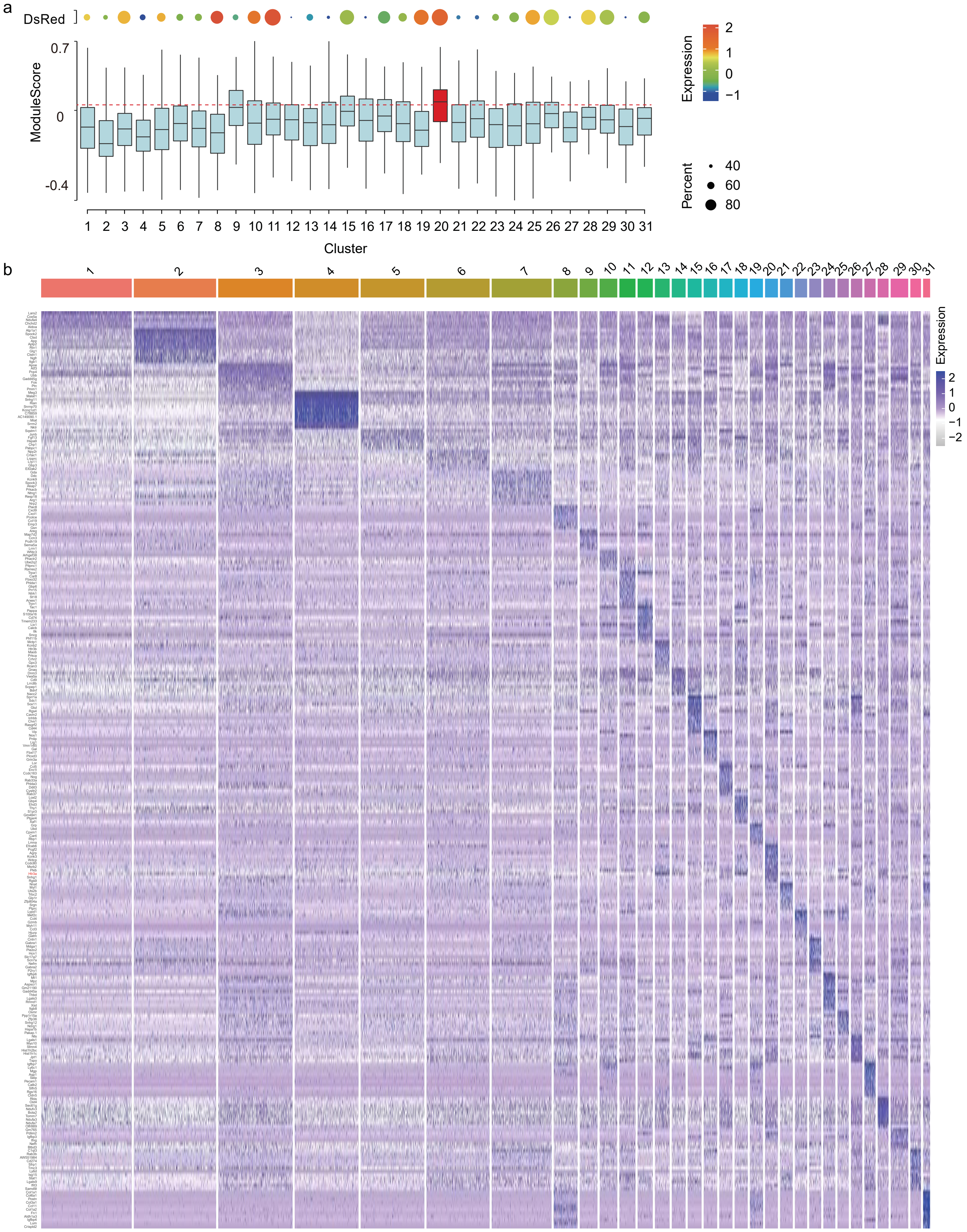
a
DsRed
ModuleScore
0.7
0
-0.4
1 2 3 4 5 6 7 8 9 10 11 12 13 14 15 16 17 18 19 20 21 22 23 24 25 26 27 28 29 30 31
Cluster
Expression
2
1
0
−1
Percent
40
60
80
b
1 2 3 4 5 6 7 8 9 10 11 12 13 14 15 16 17 18 19 20 21 22 23 24 25 26 27 28 29 30 31
Expression
2
1
0
−1
−2

Figure S13

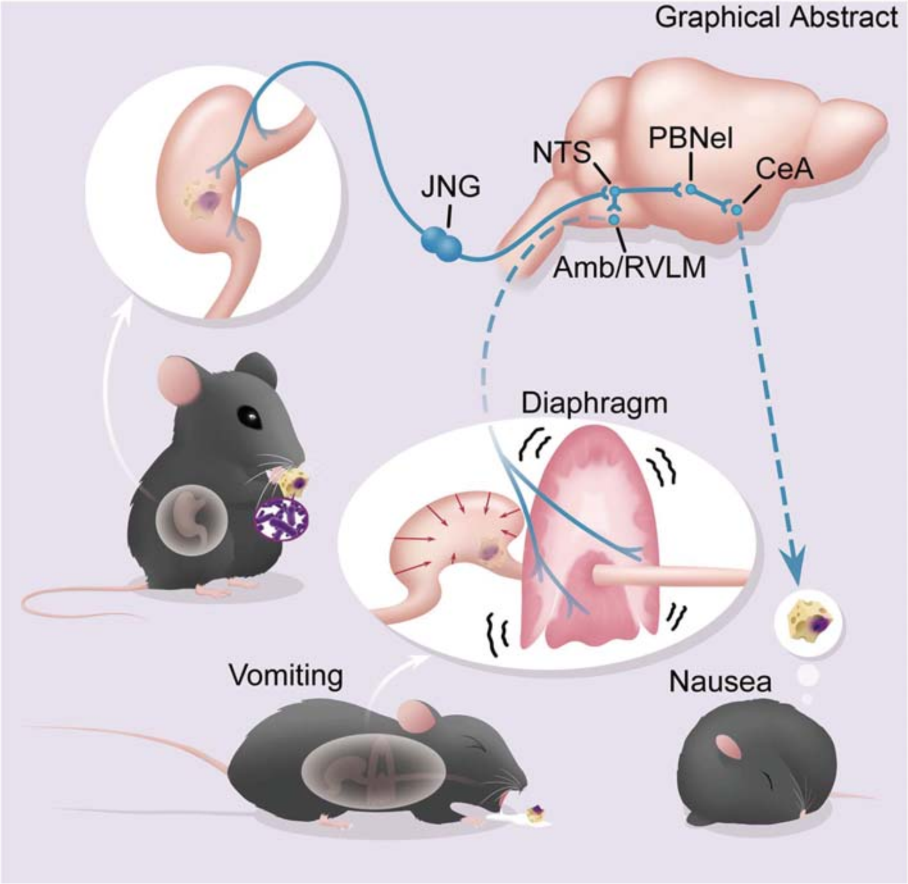
Graphical Abstract
NTS
PBNel
CeA
JNG
Amb/RVLM
Diaphragm
Vomiting
Nausea

Figure S14
